# Development of 2-D and 3-D culture platforms derived from decellularized nucleus pulposus

**DOI:** 10.3389/fbioe.2022.937239

**Published:** 2022-09-27

**Authors:** Marco A. Herrera Quijano, Nadia Sharma, Pascal Morissette Martin, Cheryle A. Séguin, Lauren E. Flynn

**Affiliations:** ^1^ Department of Anatomy and Cell Biology, Schulich School of Medicine and Dentistry, The University of Western Ontario, London, ON, Canada; ^2^ Bone and Joint Institute, The University of Western Ontario, London, ON, Canada; ^3^ School of Biomedical Engineering, The University of Western Ontario, London, ON, Canada; ^4^ Department of Physiology and Pharmacology, Schulich School of Medicine and Dentistry, The University of Western Ontario, London, ON, Canada; ^5^ Department of Chemical and Biochemical Engineering, Faculty of Engineering, The University of Western Ontario, London, ON, Canada

**Keywords:** intervertebral disc (IVD) degeneration, nucleus pulposus, decellularization, extracellar matrix, coatings, hydrogel composite, methacrylated chondroitin sulphate, cell culture models

## Abstract

Bioscaffolds derived from the extracellular matrix (ECM) have shown the capacity to promote regeneration by providing tissue-specific biological instructive cues that can enhance cell survival and direct lineage-specific differentiation. This study focused on the development and characterization of two-dimensional (2-D) and three-dimensional (3-D) cell culture platforms incorporating decellularized nucleus pulposus (DNP). First, a detergent-free protocol was developed for decellularizing bovine nucleus pulposus (NP) tissues that was effective at removing cellular content while preserving key ECM constituents including collagens, glycosaminoglycans, and the cell-adhesive glycoproteins laminin and fibronectin. Next, novel 2-D coatings were generated using the DNP or commercially-sourced bovine collagen type I (COL) as a non-tissue-specific control. In addition, cryo-milled DNP or COL particles were incorporated within methacrylated chondroitin sulphate (MCS) hydrogels as a 3-D cell culture platform for exploring the effects of ECM particle composition. Culture studies showed that the 2-D coatings derived from the DNP could support cell attachment and growth, but did not maintain or rescue the phenotype of primary bovine NP cells, which de-differentiated when serially passaged in monolayer culture. Similarly, while bovine NP cells remained highly viable following encapsulation and 14 days of culture within the hydrogel composites, the incorporation of DNP particles within the MCS hydrogels was insufficient to maintain or rescue changes in NP phenotype associated with extended *in vitro* culture based on gene expression patterns. Overall, DNP produced with our new decellularization protocol was successfully applied to generate both 2-D and 3-D bioscaffolds; however, further studies are required to assess if these platforms can be combined with additional components of the endogenous NP microenvironment to stimulate regeneration or lineage-specific cell differentiation.

## 1 Introduction

Low back pain (LBP) is the leading cause of disability worldwide (Murray et al., 2013), with a predicted lifetime prevalence of 80% (Balagué et al., 2012). While multifactorial, back pain has been associated with degeneration of the intervertebral disc (IVD) (Freemont 2009; DePalma, Ketchum, and Saullo 2011). IVD degeneration is marked by decreased IVD height, reduced nucleus pulposus (NP) hydration associated with a decrease in sulphated glycosaminoglycans (sGAGs) in the extracellular matrix (ECM), loss of the distinct NP-annulus fibrosus (AF) boundary, and fibrosis of the NP, leading to an inability of the IVD to withstand compressive loads (Roughley 2004; Inoue et al., 2012). Within the NP there is a shift towards greater ECM catabolism, cell senescence, apoptosis, inflammation, and in-growth of vasculature and nociceptive nerves (Freemont 2009).

Given the lack of disease-modifying interventions for disc degeneration, research has focused on engineering regenerative strategies targeting the NP (Yang and Li 2009; Fiordalisi et al., 2020). While cell therapies have shown promise (Schol and Sakai 2019), long-term cell survival may be impacted by the harsh microenvironment within the degenerated NP, limiting their therapeutic efficacy (Ludwinski et al., 2013). Moreover, direct IVD cell injections often result in cell leakage from the injection site (Vadalà et al., 2012). Cell encapsulation within biomaterial scaffolds offers a potential solution to support cell retention, survival and function *in vivo* by protecting the cells from the adverse inflammatory microenvironment associated with IVD degeneration (Drury and Mooney 2003; O’Halloran and Pandit 2007). Considerable research has focused on exploring the effects of various cell delivery platforms on regenerative cell populations (Drury and Mooney 2003; Gruber et al., 2004; O’Halloran and Pandit 2007; Tang et al., 2020), including studies suggesting that tissue-specific ECM can be harnessed to direct the lineage-specific differentiation of stem or progenitor cells (Lin et al., 2016; Robb et al., 2018; Zhou et al., 2018).

To fabricate ECM-derived bioscaffolds, decellularization techniques have been developed to remove immunogenic cellular components from tissues, while striving to preserve the native ultrastructure and cell-instructive biochemical composition of the ECM (Crapo et al., 2011). Various groups have reported NP decellularization protocols that can extract cellular content to obtain scaffolds enriched in structural ECM components (Wachs et al., 2017; Piening et al., 2022; Illien-Jünger et al., 2016; Mercuri, Gill, and Simionescu 2011; Fernandez et al., 2016; Xu et al., 2019; Chan et al., 2013). Despite its low cellularity, decellularization of the NP is challenging because it contains a high concentration of GAGs that are more easily extracted during processing as compared to highly crosslinked collagens (Tsuchiya et al., 2014; Peloso et al., 2016; Hanai et al., 2020). To date, the published NP decellularization protocols involve treatment with various types of detergents including Triton X-100, Triton X-200, sodium deoxycholate and sodium dodecyl sulphate (SDS). Notably, detergents can affect protein structure and have been reported to cause loss of GAGs and growth factors from a variety of tissues (Gilbert et al., 2006; Gilpin and Yang 2017). In addition, residual detergent can potentially have cytotoxic effects if not adequately cleared at the end of processing (Crapo et al., 2011). As such, our initial goal was to establish a detergent-free protocol for the decellularization of bovine NP that preserved collagens and GAGs.

Applying decellularized tissues in their intact form offers limited flexibility in terms of the scaffold properties. As such, there is great interest in the development of more customizable ECM-derived scaffold formats that incorporate the tissue-specific ECM composition within platforms that enable greater tuning of cell-cell and cell-ECM interactions. Using decellularized adipose tissue (DAT) as an ECM source, our team has previously established methods for fabricating novel ECM-derived coatings, microcarriers, foams, and hydrogel composites (Cheung et al., 2014; Kornmuller et al., 2017; Yu et al., 2017; Shridhar et al., 2019; Shridhar et al., 2020). Importantly, we demonstrated that the pro-adipogenic properties of the DAT in directing the differentiation of human adipose-derived stromal cells (ASCs) were conserved in these various platforms (Turner et al., 2012; Yu et al., 2013; Cheung et al., 2014; Shridhar et al., 2019). Supporting the potential of ECM-derived bioscaffolds in the context of IVD regeneration, Wachs et al. (2017) used pepsin digestion to generate decellularized nucleus pulposus (DNP) hydrogels capable of *in situ* gelation, which promoted human NP cell viability and GAG production. In another approach, Zhou et al. (2018) combined micronized porcine DNP with chondroitin sulphate to counteract the loss of GAGs observed following detergent-based decellularization and crosslinked the materials with genipin to create an injectable DNP-based cell delivery system. Encapsulation of human ASCs within the composite DNP-based material was reported to induce NP-like differentiation and the synthesis of aggrecan and type II collagen (Zhou et al., 2018).

Building from this foundation and using our approaches previously developed for adipose tissue, we incorporated the DNP generated with our new detergent-free decellularization protocol within 2-D coatings and 3-D methacrylated chondroitin sulphate (MCS) hydrogels. MCS was selected as it provides a cell-supportive matrix (Cheung et al., 2014; Shridhar et al., 2019) mimicking the proteoglycan-rich ECM of the native NP, in which aggrecan incorporating a high content of chondroitin sulphate side chains is the most abundant proteoglycan (Collin et al., 2017). We subsequently tested these platforms for their effects on primary bovine NP cell viability and phenotype *in vitro* in comparison to controls generated with commercially-sourced collagen type I to assess whether the tissue-specific ECM could help to maintain or restore the differentiated phenotype of the NP cells in culture.

## 2 Methods

### 2.1 Materials

Unless otherwise stated, all reagents were purchased from Sigma Aldrich Canada Ltd. and used as received (Oakville, Canada).

### 2.2 Bovine nucleus pulposus decellularization

Bovine tails were acquired from female cows (18–24 months of age) at the Mount Brydges Abattoir within 2 h post-mortem. Tails were stored at −20°C until use and then thawed overnight at 4°C prior to dissection. The musculature and soft connective tissues surrounding the vertebrae were removed under aseptic conditions. The nucleus pulposus was isolated from the IVDs of the five to six most proximal motion segments from each tail and placed in sterile 1x phosphate-buffered saline (PBS). Pooled NP tissues were minced into 2 mm^3^ pieces using a scalpel and 2 mm biopsy punch.

**FIGURE 1:**
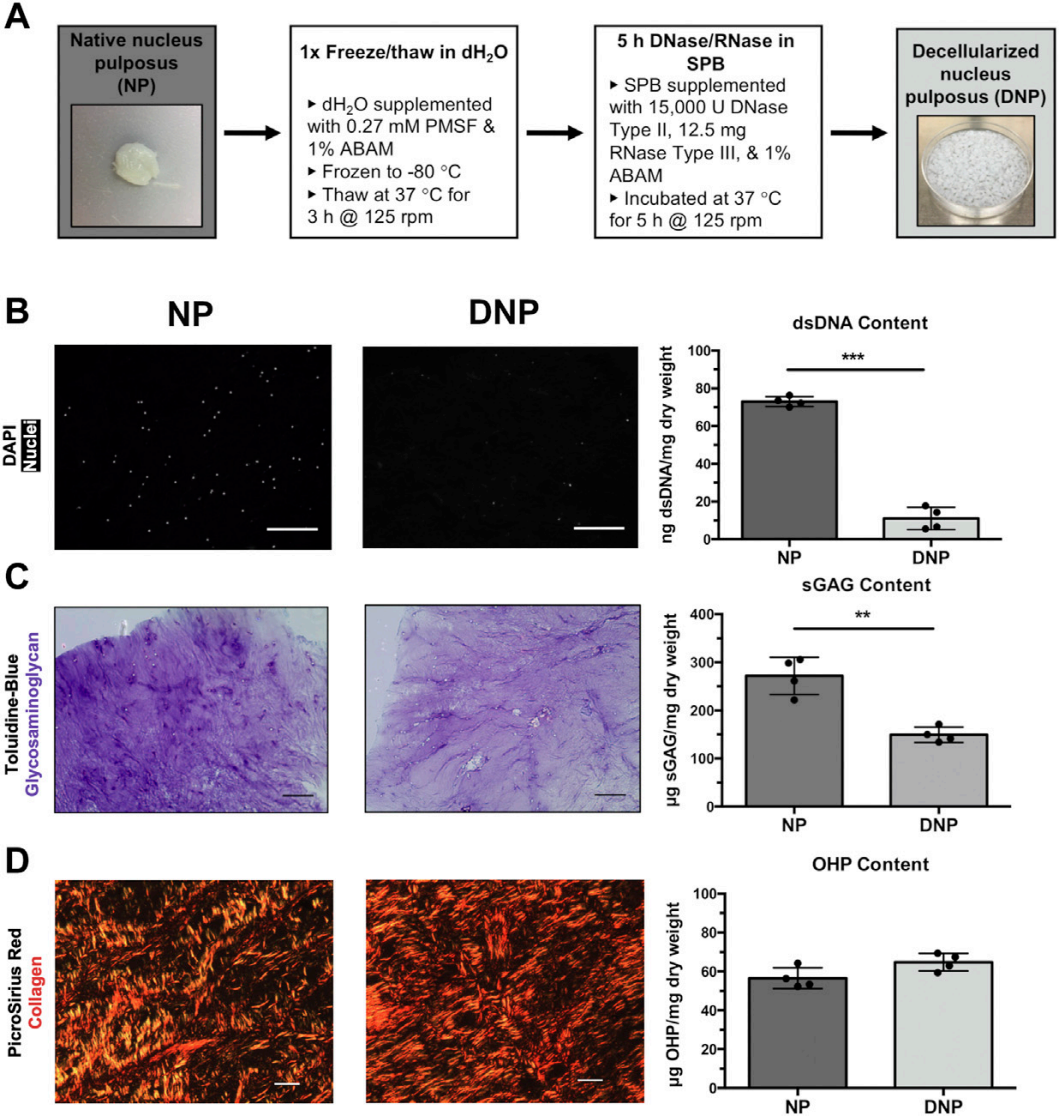
Detergent-free NP decellularization protocol effectively extracts cellular content while retaining sulphated glycosaminoglycan (sGAG) and collagen content from native NP. **(A)** Schematic of detergent-free decellularization protocol. **(B)** Representative DAPI stained sections of native nucleus pulposus tissue (NP) and decellularized NP (DNP) showed a reduction in cell nuclei (pseudo-coloured in white) following decellularization. Scale bars = 200 μm. PicoGreen quantification showed a significant reduction in dsDNA content in the DNP relative to the native NP. **(C)** Representative histological sections of NP and DNP stained with Toluidine blue detected GAGs (purple) throughout both DNP and native NP tissue sections, although staining intensity appeared to be reduced within DNP relative to NP. DMMB analysis showed ∼45% retention of sGAG content within the DNP relative to the native NP. **(D)** Representative PicroSirus Red staining of NP and DNP imaged by polarized light microscopy. Staining revealed a complex network of collagen fibers, with similar patterns detected in the DNP and native NP tissues. Hydroxyproline (OHP) quantification as a measure of total collagen content showed no significant difference between DNP and native NP tissues. Scale bars = 200 μm. Data reported as mean ± SD (N = 4), **p < 0.001, ***p < 0.0001.

For decellularization, 100 ml of solution was used per ∼15 g starting mass of NP, with all processing steps performed at 37°C under agitation on an orbital shaker (125 rpm). All decellularization solutions were supplemented with 1% antibiotic-antimycotic (ABAM) (Gibco®, Invitrogen, Burlington, Canada) solution and 0.27 mM of the protease inhibitor phenylmethylsulfonyl fluoride (PMSF). The minced NP was frozen at −80°C in deionized water (dH_2_O) overnight and subsequently thawed at 37°C over 3 h to promote cell lysis. The tissues were then enzymatically digested for 5 h with 15,000 U DNase Type II (from bovine pancreas) and 12.5 mg RNase Type III (from bovine pancreas) in Sorensen’s phosphate buffer digest solution (SPB digest) comprised of 0.17 M KH_2_PO_4_, 0.55 M Na_2_HPO_4_·7H_2_O, and 0.049 M MgSO_4_·7H_2_O, (pH 7.3). The resultant decellularized nucleus pulposus (DNP) samples were washed three times for 30 min in PBS prior to freezing at −80°C in dH_2_O and lyophilization for 72 h (Figure 1A). NP tissues from six tails were combined to generate a single batch of DNP for subsequent use in all studies.

### 2.3 Preparation of DNP/COL particles and coatings

DNP and commercially-sourced bovine type I collagen (COL) (Advanced Biomatrix, Cat # 5164-5GM, Carlsbad, United States) were cryo-milled for synthesis of DNP and COL particles using previously-established methods (Shridhar et al., 2020, 2019). In brief, cryo-milling was performed by transferring 1 g of lyophilized DNP or COL into a 25 ml milling chamber with two 10 mm stainless steel milling balls. The chamber was sealed, submerged in liquid nitrogen for 3 min, then milled for 3 min at 30 Hz (Retsch Mixer Mill MM 400 milling system). This cycle was repeated a total of three times. Cryo-milled particles were sieved through a 125 μm stainless steel mesh, and the particles that passed through the filter were collected and stored in a desiccator until further use.

To generate the ECM suspensions used to fabricate the coatings, the DNP or COL particles were added at a concentration of 25 mg/ml to 0.3% (w/w) α-amylase in 0.22 M NaH_2_PO_4_ (pH 5.4). The samples were digested under continuous agitation on an orbital shaker at 300 rpm for 72 h at room temperature, followed by centrifugation at 1,500 × *g* for 10 min. The digested DNP or COL pellet was rinsed twice with 10 ml of 5% (w/v) NaCl solution and once in dH_2_O under agitation (300 rpm) at room temperature for 10 min, and then resuspended in 0.2 M acetic acid at a concentration of 25 mg/ml (based on the initial dry mass). The samples were incubated under agitation at 120 rpm overnight at 37°C and then homogenized using a Polytron^®^ benchtop homogenizer (Thomas Scientific, Swedesboro, United States) to generate a uniform suspension, which was stored at 4°C until further use.

Working under aseptic conditions within a biological safety cabinet, the suspensions were applied to coat the surface of tissue culture plastic (TCP) multi-well plates (125 μl/cm^2^) and left to dry overnight. Prior to culture, the coatings were decontaminated by rinsing three times in fresh 70% ethanol diluted with sterile dH_2_O for 30 min, and then rehydrated by incubation for 30 min in 35% ethanol, followed by three rinses in sterile PBS and two rinses in complete NP media [DMEM supplemented with 10% fetal bovine serum (FBS) and 1% penicillin/streptomycin (P/S)] prior to cell seeding.

### 2.4 Preparation of composite MCS ± DNP/COL hydrogel scaffolds

Composite methacrylated chondroitin sulphate (MCS) hydrogels incorporating DNP or COL particles were synthesized using previously-established photo-polymerization methods (Cheung et al., 2014). MCS was first synthesized through the methacrylation of chondroitin sulphate (50 kDa, LKT Laboratories Inc., St. Paul, United States) with methacrylate anhydride, following previously established protocols (Shridhar et al., 2017), to obtain a final degree of methacrylation of 17%, as confirmed by ^1^H NMR spectroscopy on an Inova 600 NMR spectrometer (Varian, United States) (Supplementary Figure 1).

MCS was added to PBS (for mechanical testing) or dH_2_O (for gel content analysis) at a concentration of 20% (w/v). The sieved cryo-milled DNP or COL particles were then added to the MCS pre-polymer solution at a concentration of 5% (w/v), selected based on pilot studies that demonstrated it was the highest concentration that could be reproducibly added without interfering with hydrogel crosslinking. The MCS pre-polymer solution was left to dissolve overnight under agitation (100 rpm) at 37°C. Immediately prior to crosslinking, Irgacure 2959 photo-initiator was dissolved in PBS (5 mg/ml) for 2.5 h, sterile filtered, and incorporated into the MCS pre-polymer solution at a final concentration of 0.05% (w/v). The pre-gel solutions were then transferred into 1 ml syringe molds (ThermoFisher Scientific Inc.), and photo-crosslinked for 2 min on each side (4 min total) through exposure to long-wavelength ultraviolet light (365 nm, intensity of 12 mW/cm^2^).

### 2.5 DNP characterization

#### 2.5.1 Histology

To confirm decellularization and assess the presence and distribution of glycosaminoglycans (GAGs) and collagen, DNP and native NP tissue samples were fixed, paraffin-embedded, and sectioned at a thickness of 7 μm, prior to staining with either 4′,6-diamidino-2-phenylindole (DAPI), Toluidine blue (0.1%, Sigma), or Picrosirius red (1.3% picric acid and 0.1% direct red 80 (Sigma)), using previously established methods (Shridhar et al., 2019, 2020). DAPI staining was visualized using an EVOS FL fluorescence microscope (ThermoFisher Scientific Inc.), and Toluidine blue and PicroSirius Red staining were visualized using an EVOS XL Core microscope (ThermoFisher Scientific Inc., Burlington, Canada) and a Nikon Optiphot polarizing microscope (Nikon Instruments Inc., Melville, United States), respectively.

#### 2.5.2 Immunohistochemical characterization of ECM composition

Immunohistochemical (IHC) staining was performed to assess the presence and distribution of ECM components within the minced DNP samples relative to native NP tissue controls. In brief, the samples were fixed and embedded in Tissue-Tek OCT compound (Sakura Finetek, Torrance, United States), snap frozen in liquid nitrogen, and cryo-sectioned (7 μm transverse sections). Tissue sections were fixed in acetone, blocked in 10% goat serum in Tris-buffered saline supplemented with 0.1% Tween (TBST), and incubated for 1 h at 4°C with primary antibodies diluted in TBST with 2% BSA against: collagen type I (1:100, ab34710, Abcam, Toronto, Canada), collagen type II (1:200, ab34712, Abcam), collagen type V (1:300, ab7046, Abcam), collagen type VI (1:300, ab6588, Abcam), fibronectin (1:150, ab23750, Abcam), laminin (1:200, ab11575, Abcam), or keratan sulphate (1:200, sc73518, Santa Cruz, Biotechnology). Detection was carried out with either a goat anti-rabbit IgG secondary antibody conjugated to Alexa Fluor 594 (1:200, ab150080, Abcam) or a goat anti-mouse IgG secondary antibody conjugated to Alexa Fluor 680 (1:200, ab175775, Abcam), in 2% BSA in TBST for 1 h at room temperature. Slides were mounted in fluoroshield mounting medium (Abcam). No primary antibody controls were included for all experiments. Images were acquired using a EVOS FL fluorescence microscope (Thermo Fisher Scientific Inc.).

#### 2.5.3 Biochemical characterization of DNP and COL platforms

For biochemical analyses to quantitatively assess cell content, as well as retention of sulphated GAG (sGAG) and collagen content following decellularization, DNP and native NP samples were lyophilized and cryo-milled as described above without sieving, and then incubated in a digestion buffer comprised of 100 nM Na_2_HPO_4_, 5 mM EDTA, and 5 mM L-Cysteine in dH_2_O (pH 6.5) supplemented with 125 μg/ml of papain at 60°C for ∼18 h. dsDNA content was quantified using the Quant-iT™ PicoGreen^®^ dsDNA kit (Invitrogen) according to the manufacturer’s instructions. sGAG and collagen content were quantified using the dimethylmethylene blue (DMMB) and hydroxyproline (OHP) assays, respectively, based on published methods (Martin et al., 2019). The resulting data was normalized to the initial dry mass of each sample. In addition, the DMMB and OHP assays were performed following similar methods to quantify the sGAG and collagen content in the sieved DNP and COL particles, as well as the DNP and COL coatings.

#### 2.5.4 SEM analysis of DNP and COL particle and coating ultrastructure

SEM analysis was used to visualize the ultrastructure of the sieved and lyophilized DNP and COL particles, as well as the DNP and COL coatings, using established protocols (Shridhar et al., 2020). To visualize their interior structure, the DNP and COL coatings were scratched using a scalpel blade and then lyophilized. Briefly, the dry samples were coated with osmium prior to imaging with a LEO 1530 microscope at an accelerating voltage of 1–2 kV and working distance of 4–6 mm.

#### 2.5.5 DNP and COL particle size analysis

To determine their particle size distributions, cryo-milled and sieved DNP and COL particle samples were analyzed using a Malvern Mastersizer 2000 (Malvern Instruments Ltd., Worcestershire, United Kingdom). In brief, ∼300 mg samples of the particles were rehydrated in dH_2_O and analyzed according to manufacturer’s instructions (Brown and Flynn. 2015).

### 2.6 Composite hydrogel characterization

Gel content analysis of the MCS ± DNP/COL hydrogels was performed to quantify the amount of the MCS pre-polymer that was incorporated into the hydrogel network and confirm that the ECM particles were not interfering with the crosslinking efficiency, following published protocols (Cheung et al., 2014).

In addition, unconfined compression testing was performed to compare the bulk compressive properties of the MCS ± DNP/COL hydrogels. To generate consistent scaffolds sized for mechanical testing, 110 μl cell-free single-phase (MCS only) and composite MCS + DNP/COL hydrogels were synthesized and photo-crosslinked individually within the syringe molds. Following photo-crosslinking, the hydrogels were equilibrated overnight in PBS at 37°C. Immediately prior to mechanical testing, the height and diameter of the hydrogels were measured using digital calipers, to confirm the samples had a height to diameter ratio of ∼1.5. Unconfined compression testing was carried out using a UniVert system (CellScale Biomaterials Testing, Waterloo, Canada) fitted with a 0.5 N load cell and a PBS bath maintained at 37°C. Samples were subjected to two pre-conditioning cycles to a maximum of 10% strain at a rate of 0.05%/s. The hydrogels then underwent four cycles of cyclic compression testing with a 0.01 N preload applied at the beginning of each cycle, and a maximum strain of 10% at a rate of 0.05%/s per cycle (Shridhar et al., 2019). Young’s moduli were calculated from the slope of the linear region of the nominal stress-strain curve, with the stress values at 7% and 10% strain being used as the boundary conditions.

### 2.7 2-D and 3-D cell culture studies

#### 2.7.1 Primary bovine NP cell isolation and culture

Primary NP cells were isolated from bovine IVD tissues using previously published methods (Séguin et al., 2004). For each cell isolation, NP tissues were pooled from the five to six most proximal motion segments from two tails (female cows, 18–24 months of age). Isolated NP cells were seeded at an initial density of 25,000 cells/cm^2^ onto TCP dishes (ThermoFisher Scientific Inc.) and maintained in complete NP media (DMEM, 10% FBS, 1% P/S) at 37°C and 5% CO_2_. Cells seeded directly following isolation were designated as passage 0 (P0). Culture media was changed every 2 days and the cells were passaged at 85%–90% confluence (approximately 5 days).

#### 2.7.2 Cell seeding/passaging on the DNP/COL coatings

Culture studies were performed using the DNP and COL coatings to assess whether culturing on the tissue-specific ECM could help to maintain the phenotype of NP cells serially passaged in monolayer culture. For these studies, NP cells (P0) were seeded at a density of 25,000 cells/cm^2^ on the DNP coatings, COL coatings, or uncoated TCP controls and cultured until the TCP cells reached ∼85% confluence (37°C, 5% CO_2_). The NP cells cultured on the ECM coatings were passaged by digesting the DNP or COL with 0.25% collagenase A in serum-free media (DMEM supplemented with 1% P/S) for 45 min at 37°C, with pipetting every 15 min. The TCP control cells were passaged using trypsin (Gibco). The isolated cells were centrifuged at 1,200 × *g* for 5 min, washed once in complete NP media, and then resuspended in complete NP media and re-seeded on their respective coatings at 25,000 cells/cm^2^. This process was repeated to generate cells from P0 to P3 for viability and gene expression analyses, described in detail below. Time in culture to target confluence was ∼5 days for P0 cells and ∼3 days for cells between P1 and P3.

Additional studies were performed to assess the ability of the DNP coatings to rescue the phenotype of de-differentiated NP cells. For these studies, samples of the bovine NP cells were serially passaged on TCP to passage 3 (P3) to generate the de-differentiated cells. Un-passaged (P0) or de-differentiated (P3) NP cells were seeded onto the DNP coatings, COL coatings, or uncoated TCP at a density of 25,000 cells/cm^2^ and cultured for 2 or 4 days (37°C, 5% CO_2_).

#### 2.7.3 Cell encapsulation within the composite MCS hydrogels

3-D culture studies were performed to assess the effects of incorporating the tissue-specific ECM particles on the viability and phenotype of bovine NP cells encapsulated within the MCS hydrogels. Prior to cell culture, the MCS was decontaminated under UV light for 30 min and the DNP/COL particles were decontaminated in 70% ethanol overnight, followed by three successive 1 h rinses in PBS. For the studies, P0 or P3 NP cells were trypsin released, centrifuged (1,200 × *g* for 5 min) and resuspended in complete NP media at a concentration of 2 × 10^7^ cells/ml. The NP cell suspension (20% v/v) was combined with the MCS pre-polymer solution (80% v/v), the samples were well mixed with an 18G needle, and crosslinking was performed as described above. The photo-crosslinked hydrogels were extruded from the syringe molds and cut into 50 μl sized scaffolds, each containing ∼1 × 10^6^ NP cells. The hydrogels were cultured individually within low-adherence 12-well tissue culture plates (Sarstedt, Nümbrecht, Germany) in NP media at 37°C under 5% CO_2_.

### 2.8 LIVE/DEAD^®^ cell viability characterization

Confocal imaging using a LIVE/DEAD^®^ Viability/Cytotoxicity Assay (Invitrogen) was performed to assess the viability and distribution of cells on the 2-D and 3-D culture platforms, as previously reported (Shridhar et al., 2019). NP cells were imaged on the 2-D coatings or TCP controls from P0 to P3. Within the 3-D hydrogels, cells were imaged at 24 h, 72 h, 7 days, and 14 days post-encapsulation. Imaging was performed using a Zeiss LSM800 Confocal Microscope with Airyscan. Images were acquired at ×5 magnification using the tiling function and z-stack features to visualize the entire cross-section of each hydrogel at three to four depths, separated by 50 μm, starting at the surface of the gel.

### 2.9 RT-qPCR gene expression analysis

For the 2-D coating studies, the cells were collected in 1 ml PureZOL (Bio-Rad Laboratories, Mississauga, Canada) with the use of a cell scraper. For the DNP or COL coatings, the samples were sonicated three times using a Model 100 Sonic Dismembrator (ThermoFisher Scientific Inc.) in 3 s bursts. For the cells cultured within the MCS ± DNP/COL hydrogels for 24 h, 72 h, 7 days, or 14 days, four 50 μl hydrogels were pooled and placed into 2 ml of PureZOL (Bio-Rad). A microtube pestle was used to mechanical disrupt the hydrogels, followed by sonication ten times in 1 s bursts with the Model 100 Sonic Dismembrator.

Total RNA was extracted with the Aurum Total RNA Fatty and Fibrous Tissue kit (Bio-Rad), according to the manufacturer’s instructions. RNA concentration was determined using a NanoDrop 2000 spectrophotometer (ThermoFisher Scientific Inc.). Complimentary DNA was synthesized from 300 ng of RNA for the coating experiments, and 500 ng of RNA for the 3-D hydrogel experiments, using the iScript™ cDNA synthesis kit (Bio-Rad). Gene expression was analyzed by SYBR-based real-time qPCR using a Bio-Rad CFX-384 thermocycler. PCR reactions were run in triplicate, using 312 nM of forward and reverse primers, with 2x SsoFast EvaGreen Supermix (Bio-Rad). The PCR program consisted of the following: initial 2 min enzyme activation at 95°C, 10 s denaturation at 95°C, 30 s annealing/elongation at 60°C, for total of 45 cycles. Gene expression was calculated using the ΔΔCt method, normalized to expression of the housekeeping gene glyceraldehyde 3-phosphate dehydrogenase (*GAPDH*). Stability of GAPDH gene expression was validated prior to analysis (average C_q_ 22.3, min C_q_ 21.1, max C_q_ 23.5). All PCR primers were validated for efficiency and specificity, and sequences are displayed in Table 1. No template controls were included for all reactions.

**TABLE 1:** RT-qPCR list of genes and primer sequences.

Gene	Forward (5′ → 3′)	Reverse (5′ → 3′)	Primer efficiency
*COL2A1*	CCT​CTG​CGA​CGA​CAT​AAT​CT	GGT​TCT​CCT​TTC​TGT​CCC​TTT​G	91%
*COL1A1*	CCA​ATG​GCG​CTC​CTG​GTA​TT	ACC​AGG​TTC​ACC​GCT​GTT​AC	96%
*ACAN*	ACC​TAC​GAT​GTC​TAC​TGC​TAC​G	AGA​GTG​GCG​TTT​TGG​GAT​TC	106%
*SOX9*	GCA​AGC​TCT​GGA​GAC​TGC​TG	CGT​TCT​TCA​CCG​ACT​TCC​TCC	90%
*KRT 8*	CTC​CTT​CAT​CGA​CAA​GGT​GCG	CTA​TGT​TGC​TCC​GGG​CAG​T	96%
*KRT 19*	ACC​TGC​GGG​ACC​AGA​TTC​TC	AGA​CGG​GCA​TTG​TCG​ATC​TG	109%
*ACTA2*	GCC​GAG​AAC​TTT​CAG​GGA​CC	CAT​TGT​CAC​ACA​CCA​AGG​CG	98%
*FSP1* (*S100A4*)	CTT​CCT​CTC​TCT​TGC​TCC​TGA​C	GCT​TGA​ACT​TGT​CAC​CCT​CCT	102%
*GAPDH*	AAG​GTC​GGA​GTG​AAC​GGA​TTC	ATT​GAT​GGC​GAC​GAT​GTC​AA	94%

### 2.10 Statistical analyses

All numerical data are represented as mean ± standard deviation (SD), with replicate data points graphed individually. All statistical analyses were performed using GraphPad Prism 6 (GraphPad Software, San Diego, United States). A Kolmogorov-Smirnov test was done to compare the particle size distribution of the cryo-milled DNP and COL particles. The biochemical characterization data of DNP and NP was analyzed using a two-tailed unpaired Student’s *t*-test, while DNP and COL particles/coatings biochemical data were analyzed by one-way ANOVA. Gene expression levels of NP cells seeded on 2-D coatings or encapsulated within the 3-D hydrogels were compared between groups using two-way ANOVA with a Tukey’s post-hoc comparison of means. Differences with *p* < 0.05 were considered to be statistically significant.

## 3 Results

### 3.1 The decellularization protocol effectively extracted cells from bovine nucleus pulposus while retaining ECM components

The first aim of this study was to develop and validate a protocol for NP decellularization, with the overall goal of enhancing removal of cell content while retaining ECM components including GAGs and collagens. The initial decellularization protocol tested was based on methods established for decellularizing porcine auricular cartilage, which were adapted from the literature (Graham et al., 2016) and involved an extended 10-day process including freeze-thaw cell lysis in a hypotonic buffer, treatment with the non-ionic detergent Triton-X100, and enzymatic digestion with DNase and RNase. Although the protocol was effective at extracting cells, histological staining revealed minimal retention of GAGs at the end of processing (Supplementary Figure 2). Based on this, the protocol was systematically altered, including investigating the effects of varying buffer conditions, treatment times, and ultimately the elimination of the detergent treatment step.

In brief, the finalized two-step protocol involved treating minced bovine NP tissue with a single freeze-thaw cycle in dH_2_O to lyse cells, followed by a 5 h enzymatic digestion in DNase and RNase (Figure 1A). DAPI staining showed a marked reduction in cell nuclei relative to the native tissue controls (Figure 1B). The imaging results were corroborated by the PicoGreen assay results, which demonstrated a significant reduction of ∼85% in dsDNA content following decellularization (Figure 1B). Toluidine blue staining verified that GAGs were retained throughout the processed tissues, although the staining intensity was reduced in comparison to the native NP samples (Figure 1C). Consistent with these findings, quantification through the DMMB assay indicated ∼45% retention of sGAG content following decellularization (Figure 1C). Picrosirius red staining revealed similar networks of interwoven collagen fibers in both the DNP and native NP samples, and the hydroxyproline assay confirmed there was no significant difference in the total collagen content between the groups (Figure 1D).

**FIGURE 2:**
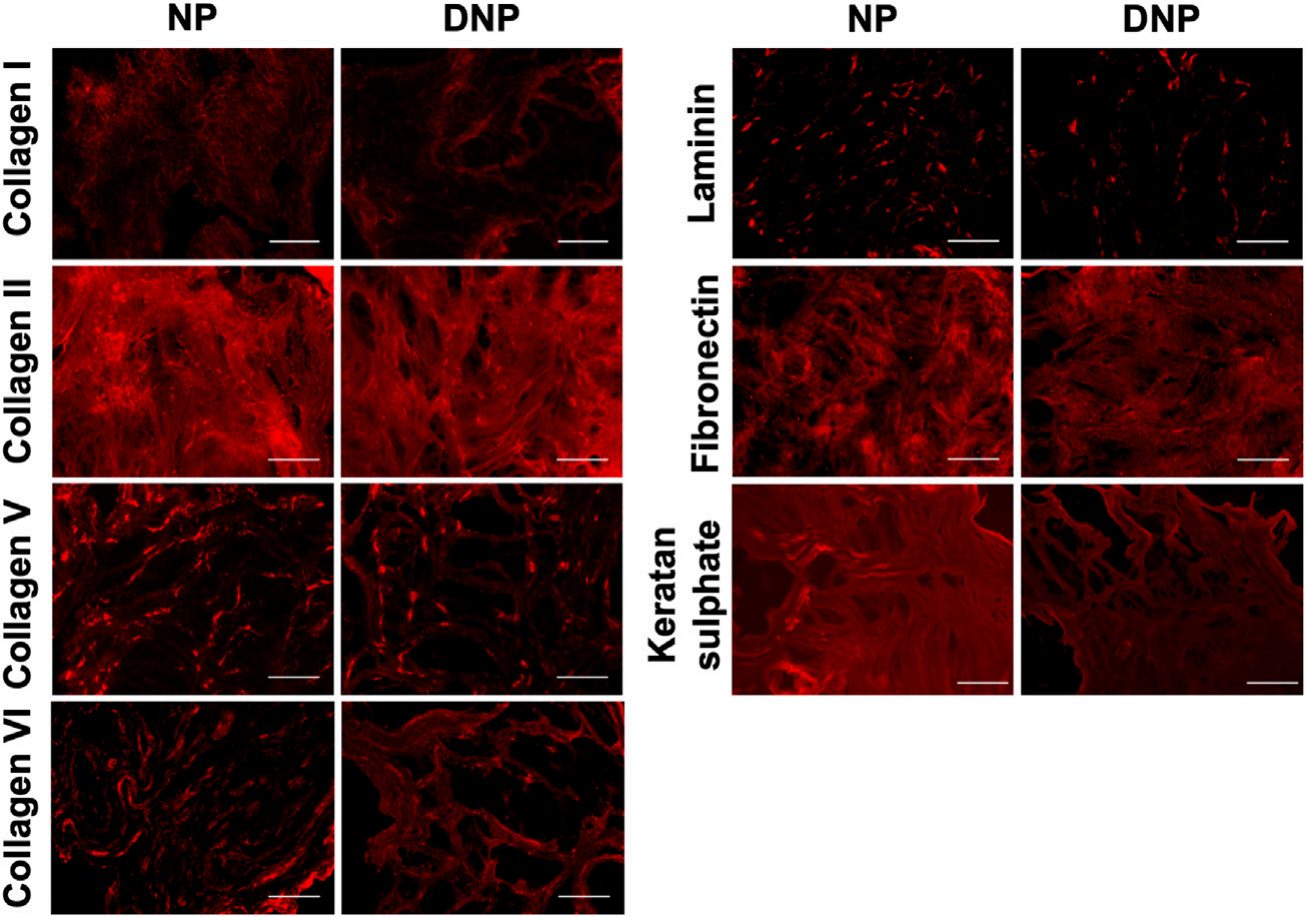
Immunofluorescence analysis confirms the maintenance of key NP ECM constituents following decellularization. Representative images showing the presence and distribution of collagen type I, collagen type II, collagen type V, collagen type VI, laminin, and fibronectin, with similar distribution patterns and staining intensities in the decellularized NP (DNP) and the native nucleus pulposus (NP). Keratan sulphate showed a similar spatial distribution pattern with qualitatively decreased staining intensity within the DNP relative to the native NP. Scale bars = 200 μm (N = 3).

Following confirmation of decellularization, immunohistochemical staining was performed to further probe the ECM composition in the DNP relative to the native NP (Figure 2). Staining for collagen types I, II, V, and VI revealed similar staining intensities and spatial distribution patterns in the DNP compared to the native NP. Similarly, both laminin and fibronectin were qualitatively well retained following decellularization. Although the spatial distribution of keratan sulphate was similar in the DNP and native NP, a decrease in staining intensity was observed following decellularization, consistent with the reduced sGAG content in the DNP.

### 3.2 Particles and coatings derived from DNP or COL are structurally and compositionally distinct

**FIGURE 3:**
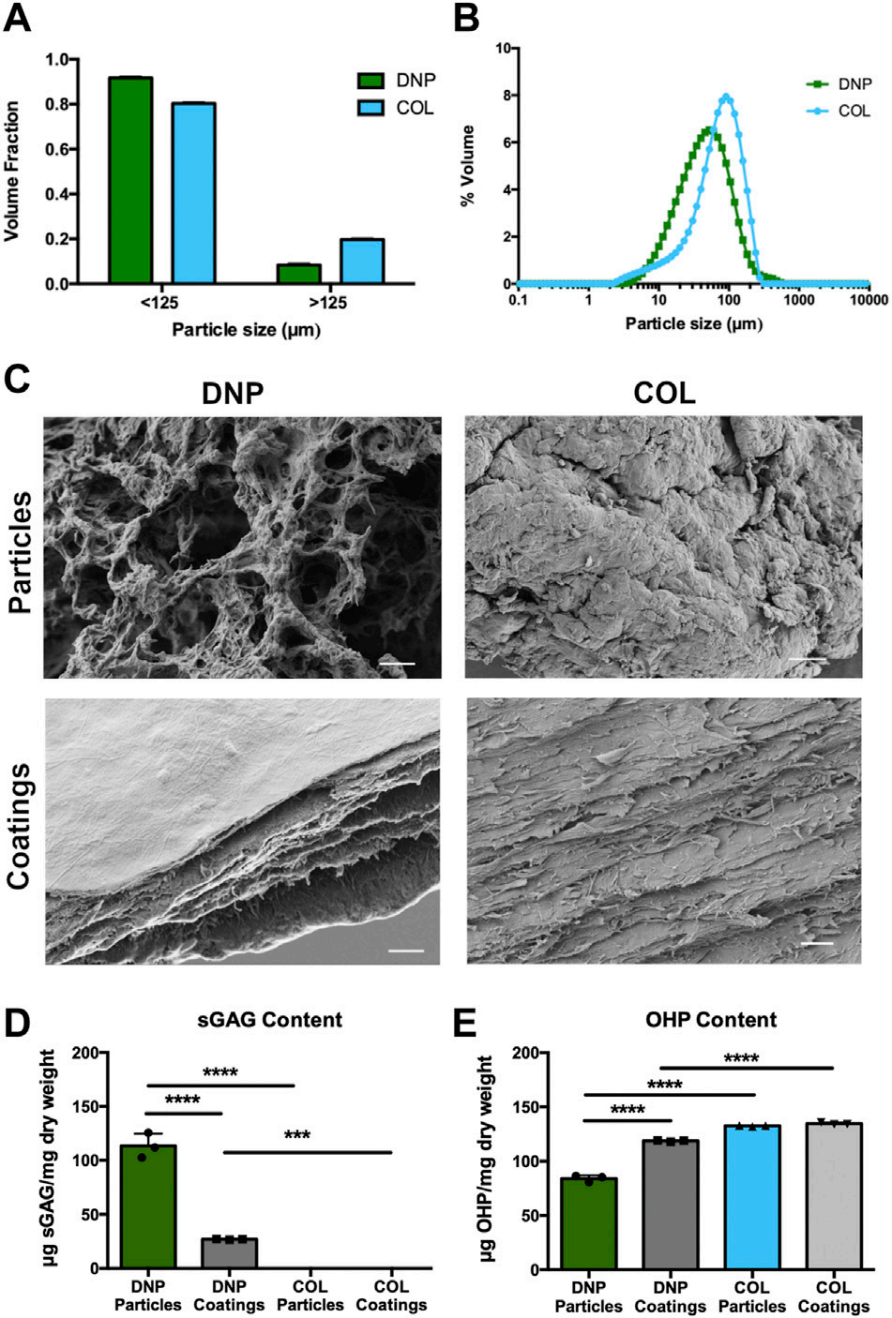
DNP particles are compositionally and structurally distinct from DNP coatings. **(A)** Volume fraction of cryomilled DNP and COL particles showed that particles were predominantly less than 125 μm (N = 3). **(B)** Particle size distribution plots based on the Mastersizer analysis. There was no significant difference between the DNP and COL groups based on the Kolmogorov-Smirnov test (*p* < 0.05). **(C)** Scanning electron microscopy (SEM) of DNP or COL particles showed a distinct extracellular matrix ultrastructure as compared to their respective coatings. Images captured at ×5,000 magnification. Scale bars = 2 μm. **(D)** DMMB analysis of sGAG content showed a significant decrease in sGAG within the DNP coatings relative to the DNP particles, while both COL particles and coatings had negligible amounts. **(E)** Collagen content as measured by the hydroxyproline (OHP) assay, showed an increase in relative collagen content within the DNP coatings as compared to the DNP particles (N = 3, ***p < 0.001, ****p < 0.0001).

Based on our findings of efficient removal of cells and general maintenance of the ECM content in the DNP generated with our detergent-free decellularization protocol, we proceeded with developing and testing 2-D and 3-D biomaterial culture platforms that could be applied to assess the effects of the DNP on the *in vitro* culture and expansion of primary bovine NP cells. Platforms were also generated using commercially-available type-I collagen sourced from bovine tendons to compare the effects of a non-tissue-specific ECM control. As a first step in fabricating both biomaterial formats, the DNP and COL were cryo-milled and sieved through a 125 μm mesh to remove larger particles. Characterization using a Mastersizer system confirmed that >80% of the DNP and COL particles were less than 125 μm (Figure 3A), with average diameters of 60.34 ± 55.44 μm for the DNP particles and 83.84 ± 54.45 μm for the COL particles. There was no significant difference in the particle size distributions between the groups based on a Kolmogorov–Smirnov nonparametric test (*p* < 0.05) (Figure 3B). To generate the coatings, applying methods previously developed by our group (Shridhar et al., 2020), the particles were subsequently digested with α-amylase and homogenized in acetic acid.

SEM analysis revealed that the DNP particles had a more open porous structure, with visible fibers observed, while the COL particles appeared more compacted with a smoother surface ultrastructure (Figure 3C). While the surface of both the DNP and COL coatings appeared smooth, imaging of the scratched region revealed that both had a multilayer internal structure, with the DNP coatings appearing to have larger gaps between the layers.

Biochemical quantification revealed a significant ∼4.2-fold reduction in relative sGAG content in the DNP coatings as compared to the DNP particles, indicating that the further processing resulted in a marked loss of GAGs (Figure 3D). No sGAG was detected in either the COL particles or coatings. In contrast, the hydroxyproline assay indicated a significant ∼1.4-fold increase in the relative collagen content in the DNP coatings as compared to the DNP particles, likely due to the dramatic reduction in GAG content observed (Figure 3E). Consistent with this interpretation, there was no significant difference in the hydroxyproline content between the COL particles and coatings.

### 3.3 DNP coatings provide a cell-supportive platform for cell expansion but do not alter the NP cell phenotype

Having generated the 2-D coating culture platform, we next assessed the effects of the ECM coatings on primary bovine NP cells over sequential passages. Since the cells on the coatings could not be visualized with brightfield microscopy, live cells were imaged using calcein-AM staining following 3 days in culture on the DNP and COL coatings, along with the TCP controls. All substrates were found to support primary NP cell attachment following serial passaging, although decreased cell density was consistently observed on the DNP and COL coatings as compared to cells cultured on TCP (Supplementary Figure 3).

**FIGURE 4:**
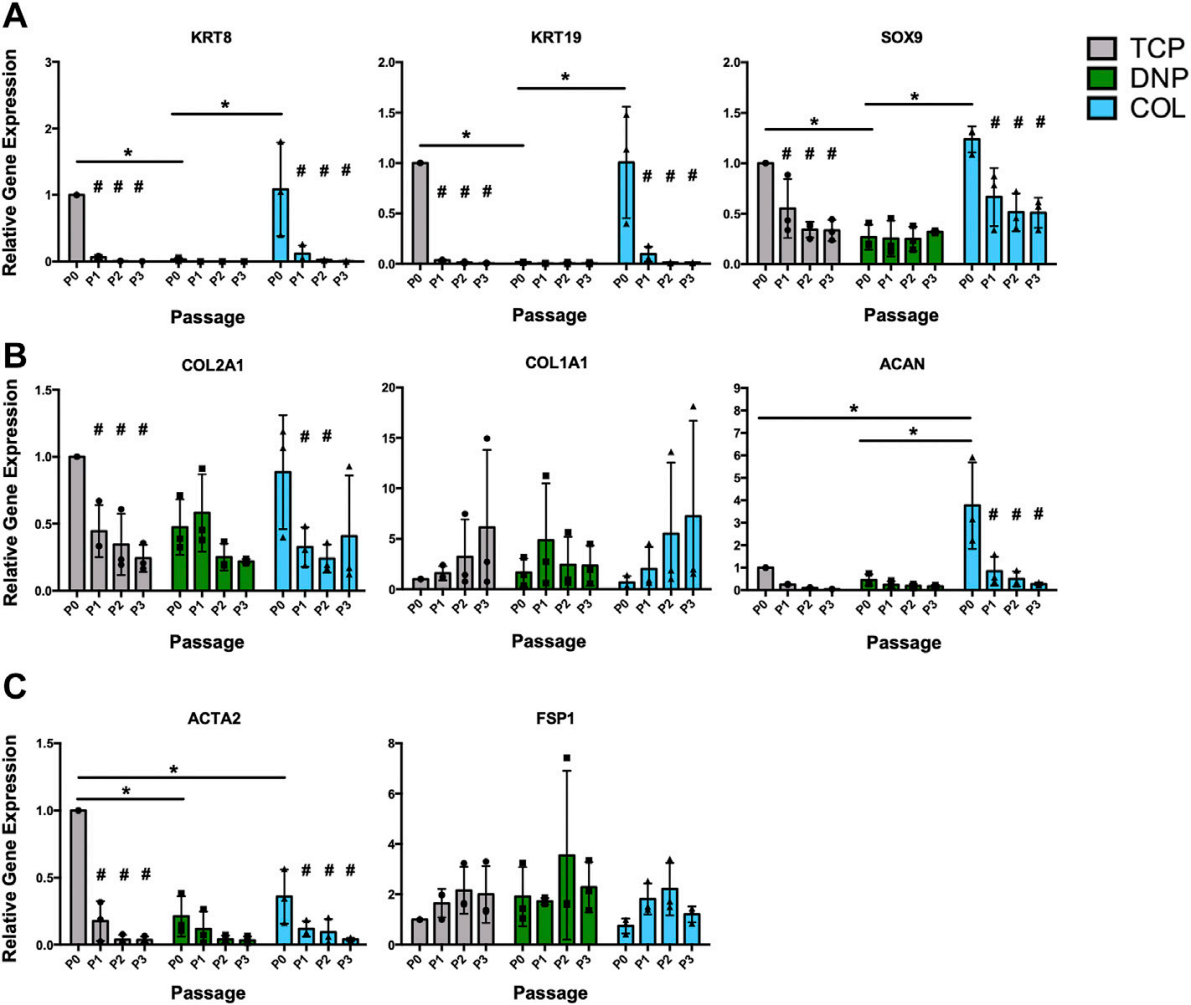
RT-qPCR gene expression analysis of serially passaged primary bovine NP cells grown in monolayer on tissue culture plastic or ECM coatings. Primary bovine NP cells (P0) were cultured in monolayer on tissue culture plastic (TCP), decellularized NP (DNP) coatings, or type I collagen (COL) coatings and serially passaged (P1-P3) on the same culture substrate. Gene expression analysis was performed to quantify expression of **(A)** NP-associated markers (*KRT8, KRT19, SOX9*), **(B)** ECM genes (*COL2A1, COL1A1, ACAN*), and **(C)** fibroblast markers (*ACTA2, FSP1*). Relative gene expression was determined using the ΔΔC_t_ method normalized to expression of the housekeeping gene *GAPDH*, and expressed relative to P0 cells on TCP. Data is presented as mean ± SD (N = 3, *p* < 0.05, * indicates significant differences between culture conditions, # indicates significant differences within each condition relative to P0 cells).

To assess the effects of culture on the 2-D ECM coatings on the maintenance of the NP cell phenotype following serial passage in monolayer culture, RT-qPCR analysis was used to quantify the expression of NP-associated markers (*KRT8, KRT19, SOX9*), ECM genes (*COL2A1, COL1A1, ACAN*), and fibroblast-associated markers (*ACTA2, FSP1*). Similar to previous reports (Cui et al., 2012; Rosenzweig et al., 2017; Ashraf et al., 2020), our analysis demonstrated a robust decrease in the expression of NP-associated markers in primary cells over serial passage (P0–P3) on TCP (Figure 4, grey bars). Compared to primary (P0) NP cells cultured on TCP, primary (P0) NP cells cultured on DNP coatings showed significantly lower expression of the NP-associated markers *KRT8*, *KRT19*, and *SOX9* - a change not induced by culture of P0 NP cells on COL coatings (Figure 4A). In general, culture of NP cells on either DNP or COL coatings did not significantly alter the expression of ECM genes compared to cells cultured on TCP, with the exception of *ACAN* which was significantly increased in P0 NP cells on COL coatings compared to P0 NP cells on either TCP or DNP coatings (Figure 4B). Lastly, although a generalized loss of NP-specific gene expression was noted in primary cells following serial passaging in 2-D on all substrates, this was not associated with increased expression of either *ACTA2*, a myofibroblast marker, or FSP1, a marker of activated fibroblasts (Figure 4C). These findings suggest that *in vitro* culture of primary bovine NP cells on DNP coatings did not promote the maintenance of the NP phenotype across serial passages when compared to cells cultured on TCP.

**FIGURE 5:**
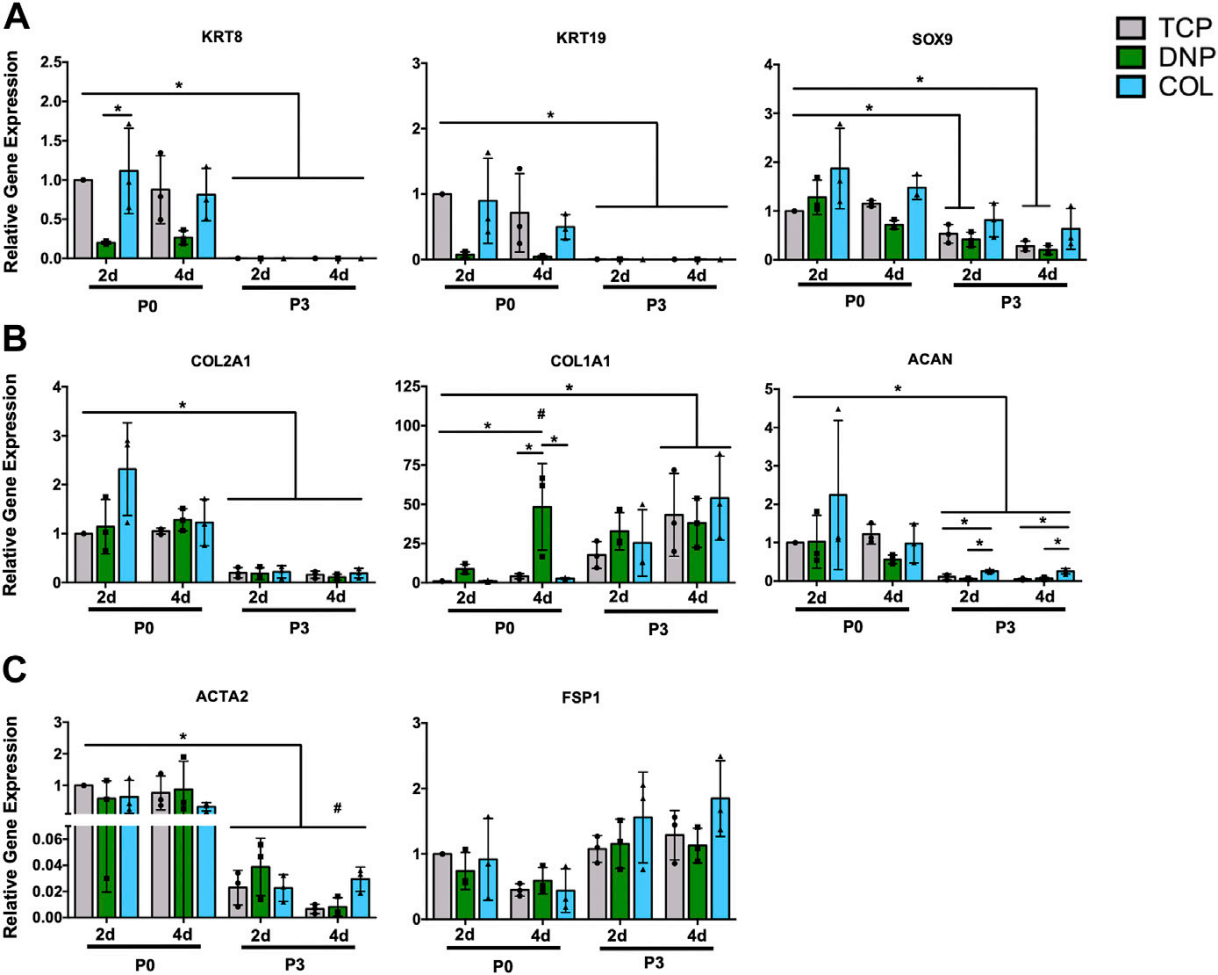
RT-qPCR gene expression analysis of primary bovine NP cells following short term culture in monolayer on tissue culture plastic or ECM coatings. Un-passaged (P0) and late passage (P3) primary bovine NP cells were seeded onto tissue culture plastic (TCP), decellularized NP (DNP) coatings, or type I collagen (COL) coatings. Cells were cultured *in vitro* for 2 days (2d) or 4 days (4d). Gene expression analysis was performed to quantify expression of **(A)** NP-associated markers (*KRT8, KRT19, SOX9*), **(B)** ECM genes (*COL2A1, COL1A1, ACAN*), and **(C)** fibroblast markers (*ACTA2, FSP1*). Relative gene expression was determined using the ΔΔC_t_ method normalized to expression of the housekeeping gene *GAPDH*, and expressed relative to P0 cells on TCP at 2 days. Data is presented as mean ± SD (N = 3, *p* < 0.05, * indicates significant difference between culture conditions, # indicates significant difference within each condition relative to 2d).

To assess the short-term effects of the ECM coatings on primary NP cells and to determine if they could restore the phenotype of de-differentiated primary NP cells, late passage NP cells (passaged three times on TCP) or primary un-passaged (P0) NP cells were seeded onto TCP, DNP coatings or COL coatings, and gene expression was assessed after 2 or 4 days. Short term culture of P0 NP cells on 2-D coatings did not significantly alter the expression of NP-associated markers or ECM genes compared to P0 cells cultured on either TCP or COL; a significant increase in the expression of *COL1A1* was however detected following 4 days of culture of P0 NP cells on DNP compared to cells cultured on TCP or COL coatings (Figures 5A,B). In general, culture of de-differentiated NP cells on ECM coatings did not reverse the loss of expression of NP markers associated with serial passage *in vitro*. The expression of *KRT8*, *KRT19*, and *COL2A1* were significantly decreased in P3 cells at both time points compared to P0 cells, unaffected by culture on the ECM coatings. This was associated with a significant increase in the expression of *COL1A1* in P3 NP cells on all coatings at 4 days compared to P0 cells (Figure 5B). The expression of *SOX9* was significantly decreased in P3 cells cultured on both TCP and DNP at both time points compared to P0 cells (Figure 5A). While the expression of *ACAN* was decreased in P3 NP cells on all substrates at both 2 and 4 days compared to P0 cells, a moderate but significant increase was detected in P3 NP cells cultured on COL compared to those cultured on either TCP or DNP (Figure 5B). Lastly, decreased expression of NP-related genes was not associated with increased expression of fibroblast-associated markers in NP cells on ECM coatings (Figure 5C). Taken together, these findings suggest that culture of de-differentiated NP cells on the DNP coatings alone was unable to rescue the NP-specific phenotype, but that the ECM source used to generate the coatings impacted NP gene expression.

### 3.4 MCS hydrogels containing DNP provide a cell supportive platform for NP cell culture but do not alter the NP cell phenotype

While culture on 2-D coatings was insufficient to maintain or rescue the phenotype of the cultured bovine NP cells, we postulated that the lack of observed bioactive effects of the DNP may be attributed to alterations in the ECM composition with the additional processing required to generate the coatings, as well as to the 2-D nature of the culture model. As such, our next step was to establish a 3-D culture platform that could be applied to encapsulate the bovine NP cells in combination with cryomilled DNP or COL particles (Supplementary Figure 4A). More specifically, we successfully fabricated composite MCS hydrogels incorporating 5% w/v ECM via UV-crosslinking that were stable in long-term culture. Gel content analysis indicated that the incorporation of the ECM did not interfere with the cross-linking process (Supplementary Figure 4B). Consistent with this interpretation, mechanical testing confirmed that there was no significant difference in the Young’s moduli between the three hydrogel groups (Supplementary Figure 4C).

**FIGURE 6:**
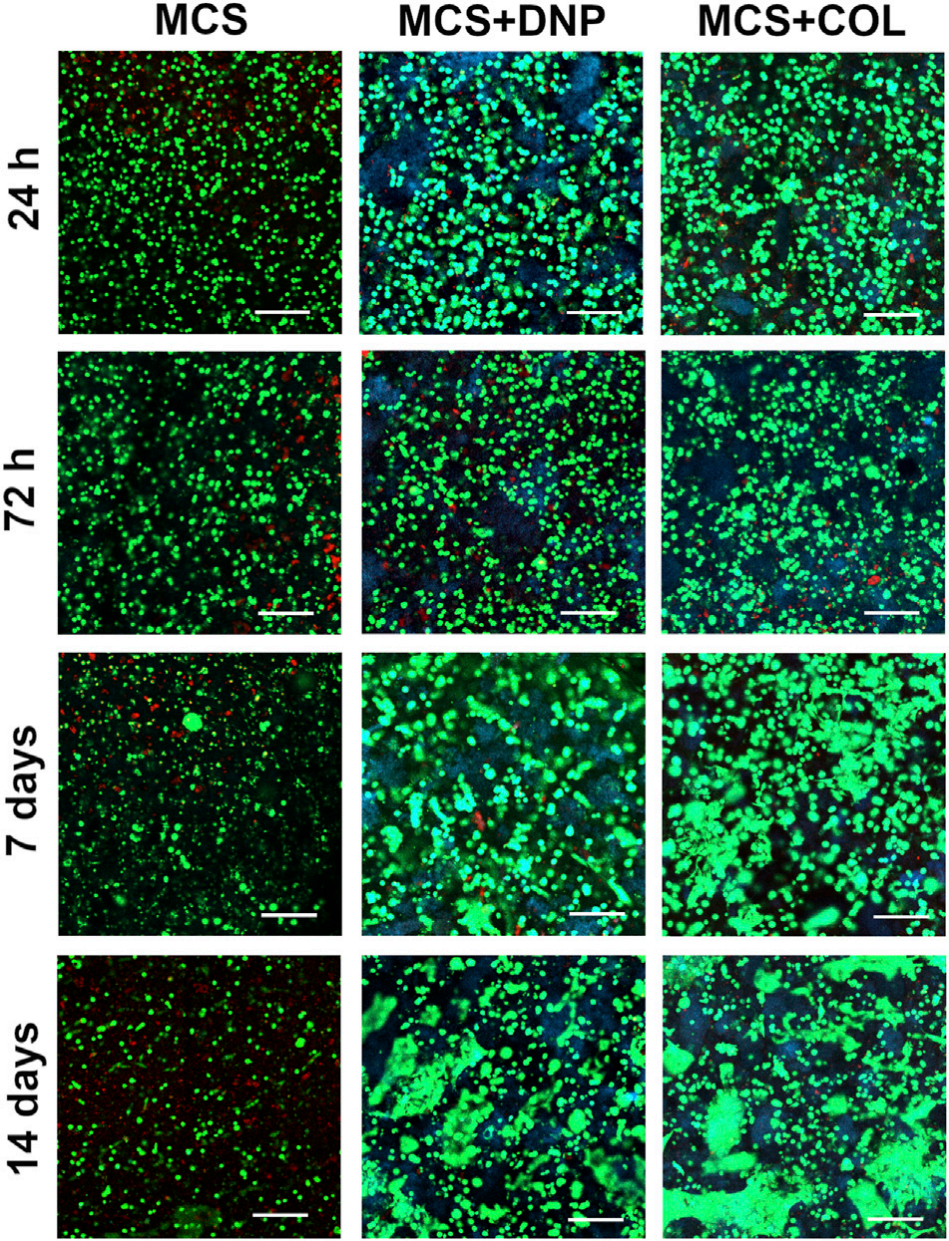
Primary bovine NP cells remain viable following encapsulation and *in vitro* culture within MCS ± DNP/COL up to 14 days. Unpassaged P0 primary bovine NP cells were encapsulated within MCS, MCS + DNP or MCS + COL hydrogels, and cultured *in vitro* for 24 h, 72 h, 7 days, or 14 days. Representative confocal microscopy images were captured at each time point showing live cells stained with calcein-AM (green), dead cells with ethidium homodimer-1 (red), and auto-fluorescence of DNP and COL particles (blue). Images were captured at depths between 75–150 μm from the surface of each hydrogel construct. Scale bars = 200 μm. Images are representative of N = 3 individual cell preparations.

Primary NP cells (P0 and P3) were encapsulated within the MCS + DNP or MCS + COL composite hydrogels, or MCS alone, to assess the capacity of the 3-D culture platforms to support NP cell viability up to 14 days in culture. Following encapsulation, P0 NP cells showed a uniform distribution throughout all hydrogels (Figure 6). In general, the MCS, MCS + DNP, and MCS + COL groups showed high cell viability across all timepoints assessed. Qualitative observations suggested a reduction in the number of viable cells present in the MCS group following 7 and 14 days of culture as compared to the MCS + DNP or MCS + COL groups. Notably, NP cell clustering was observed after 7 and 14 days in culture only within the MCS + DNP and MCS + COL hydrogels. These findings were consistent with those of P3 NP cells encapsulated within the composite hydrogels (Supplementary Figure 5), confirming that the platform supported the viability of the encapsulated bovine NP cells and suggesting that the cells were interacting with the cryomilled ECM.

**FIGURE 7:**
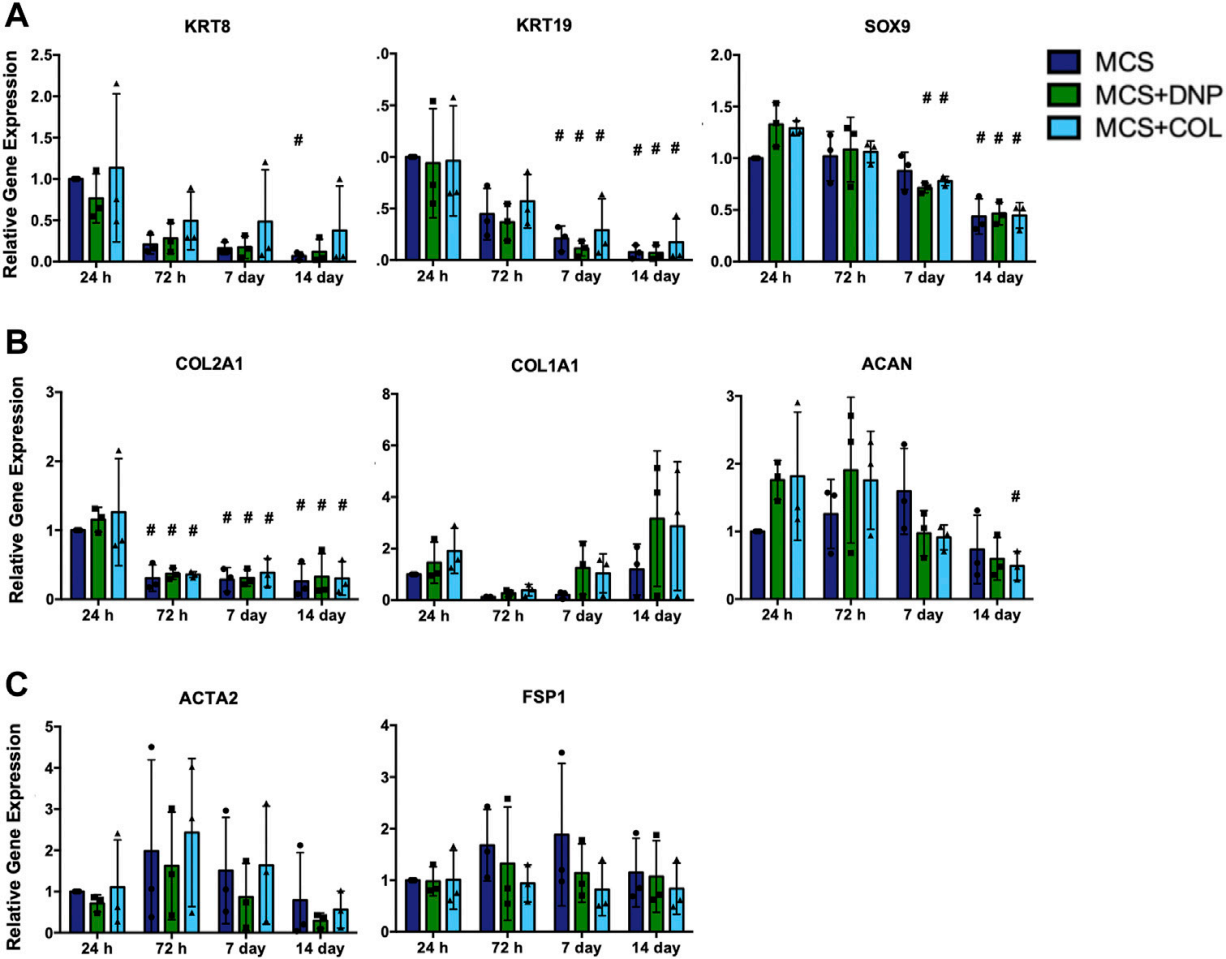
RT-qPCR gene expression analysis of primary bovine NP cells encapsulated within MCS ± DNP/COL scaffolds and cultured *in vitro* up to 14 days. Unpassaged (P0) primary bovine NP cells were encapsulated within MCS, MCS + DNP, or MCS + COL hydrogels, and cultured *in vitro* for 24 h, 72 h, 7 days, or 14 days. Gene expression analysis was performed to quantify expression of **(A)** NP-associated markers (*KRT8, KRT19, SOX9*), **(B)** ECM genes (*COL2A1, COL1A1, ACAN*), and **(C)** fibroblast markers (*ACTA2, FSP1*). Relative gene expression was determined using the ΔΔC_t_ method normalized to expression of the housekeeping gene *GAPDH*, and expressed relative to cells encapsulated within MCS at 24 h. Data is presented as mean ± SD (N = 3, *p* < 0.05, * indicates significant difference between culture conditions, # indicates significant difference within each condition relative to 24 h).

The effects of culture in the 3-D DNP-containing MCS hydrogels on the expression of NP-associated markers, ECM genes, and fibroblast-associated markers were assessed in primary un-passaged (P0) bovine NP cells up to 14 days of culture (Figure 7). Overall, the incorporation of the DNP or COL particles within MCS hydrogels did not significantly alter gene expression relative to the MCS alone at any timepoint investigated. In all 3-D cultures, primary NP cells showed a general decrease in the expression of NP-associated genes over time in culture. While a significant decrease in *ACAN* expression was only detected in MCS + COL hydrogels by 14 days in culture, a significant decrease in *KRT19*, *SOX9*, and *COL2A1* expression was detected in NP cells within all three hydrogel groups by 14 days compared to baseline gene expression in MCS (24 h) (Figures 7A,B). In keeping with our findings on 2-D coatings, the expression of the fibroblast-associated markers *ACTA2* and *FSP1* remained unchanged over time in 3-D culture (Figure 7C).

**FIGURE 8:**
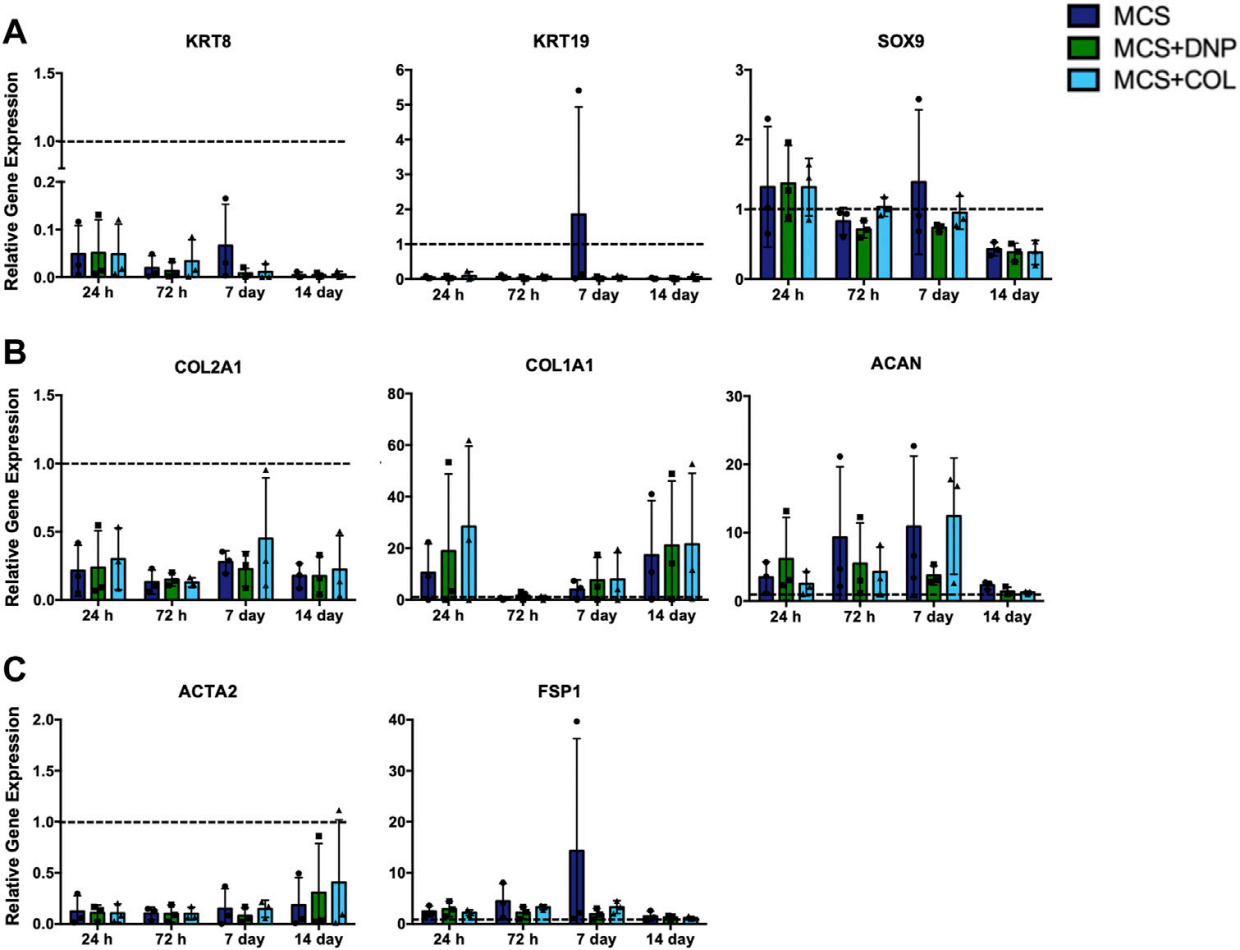
RT-qPCR gene expression analysis of late passage primary bovine NP cells encapsulated within MCS ± DNP/COL scaffolds and cultured *in vitro* up to 14 days. Late passage (P3) primary bovine NP cells were encapsulated within MCS, MCS + DNP, or MCS + COL hydrogels, and cultured *in vitro* for 24 h, 72 h, 7 days, or 14 days. Gene expression analysis was performed to quantify expression of **(A)** NP-associated markers (*KRT8, KRT19, SOX9*), **(B)** ECM genes (*COL2A1, COL1A1, ACAN*), and **(C)** fibroblast markers (*ACTA2, FSP1*). Relative gene expression was determined using the ΔΔC_t_ method normalized to expression of the housekeeping gene *GAPDH*, and expressed relative to P0 cells encapsulated within MCS at 24 h (dotted line indicated on graphs). Data is presented as mean ± SD (N = 3 *p* < 0.05).

Lastly, to assess whether culture within the 3-D MCS + DNP hydrogels could rescue the phenotype of de-differentiated NP cells, late passage (P3) NP cells were encapsulated within the MCS, MCS + DNP, or MCS + COL hydrogels and cultured up to 14 days. To contextualize changes in gene expression, these data were assessed relative to baseline levels of gene expression in P0 NP cells (MCS only at 24 h). Similar to P0 NP cells, the incorporation of the DNP or COL particles did not significantly alter gene expression in P3 NP cells relative to the MCS alone at all timepoints (Figure 8). No significant change in the expression of the NP-associated markers or ECM genes was detected over time in culture within any of the hydrogel groups (Figures 8A,B). Compared to P0 NP cells at baseline (MCS only at 24 h), P3 NP cells showed a significant reduction in the expression of *KRT8*, *COL2A1*, and *ACTA2* at all time points, unaffected by hydrogel composition. Expression of the fibroblast-associated markers *ACTA2* and *FSP1* were not altered over time in any of the hydrogel groups (Figure 8C). Taken together, these findings suggest that culture of primary NP cells within composite hydrogels containing DNP particles was not sufficient to prevent the loss of or restore the NP cell phenotype following *in vitro* cell culture.

## 4 Discussion

Many *in vitro* studies involve the expansion of cells in 2-D on rigid tissue culture plastic substrates that are convenient for supporting cell attachment and proliferation, but substantially alter cell-cell and cell-ECM interactions as compared to the native cellular microenvironment, which can markedly affect cell function (Rubashkin, Ou, and Weaver 2014; Rosenzweig et al., 2017; Ashraf et al., 2020). Notably, monolayer culture of primary bovine NP cells on TCP results in loss of the NP phenotype with serial passaging, as measured by decreased NP-associated marker expression (Ashraf et al., 2020). Recognizing the cell-instructive capacity of the ECM, the primary goal of this study was to develop 2-D and 3-D culture platforms that mimic the complex ECM within the native nucleus pulposus. As a first step, a detergent-free decellularization protocol was established that involved subjecting minced bovine NP tissue to one freeze-thaw cycle in deionized water to promote cell lysis, followed by 5 h of digestion with DNase and RNase to remove residual nucleic acids that could cause an immunogenic response if the DNP was applied *in vivo* (Gilbert et al., 2009; Gilpin and Yang 2017). Due to the limited availability of healthy human NP tissue, previous studies have similarly validated decellularization protocols using bovine or porcine NP tissues (Mercuri et al., 2011; Fernandez et al., 2016; Illien-Jünger et al., 2016; Wachs et al., 2017). The NP from these species more closely resembles the cellular and ECM composition of the human NP as compared to other preclinical animal models such as the mouse or rabbit (Demers et al., 2004; Alini et al., 2008).

In healthy tissues, the NP ECM consists predominantly of proteoglycans [∼65% of the NP ECM dry weight (Hwang et al., 2014)] including aggrecan, enmeshed within an irregular network of collagen and elastin fibers (Adams and Roughley, 2006). Chondroitin sulphate and keratan sulphate are the most abundant GAGs found within the native NP (Adams and Roughley, 2006), which contribute to a net negative charge that promotes water influx into the tissues (Roughley, 2004). The high water content of the NP [∼80% of the total wet weight (Urban and Roberts, 2003; Hwang et al., 2014)] generates swelling pressure that is critical for maintaining the IVD height and proper transmission of compressive loads (Freemont 2009; Inoue et al., 2012). GAGs are also important for the bioactivity of the ECM, sequestering growth factors and bioactive molecules to increase their local concentration (Ramirez and Rifkin, 2003), as well as directly modulating numerous cell functions including proliferation, migration, and differentiation (Peloso et al., 2016).

Decellularization of the NP is challenging because the treatments required to remove cellular content often result in a marked loss of GAGs, shifting the resultant ECM to a more collagen-rich environment that could have substantially different effects on IVD cell types. In developing our detergent-free protocol, we spent a substantial amount of time exploring the effects of various treatment steps to establish a process that was reproducibly effective and that balanced removal of cellular content with preservation of GAGs. We found that protocol duration was an important factor in GAG retention, which has also been reported with other tissue types (Gilpin and Yang, 2017; Reginensi et al., 2020), with extended protocols including additional freeze-thaw cycles having limited benefit in further reducing cell content.

Our finalized protocol resulted in an ∼85% reduction in dsDNA content, while retaining ∼45% of sGAGs based on dry weight. For comparison, Illien-Jünger et al. (2016) reported an ∼85% reduction in dsDNA content with ∼15% retention of sGAGs with their optimized protocol for decellularizing bovine NP, which involved repeated freeze-thaw cycles, followed by tissue milling and treatment with sodium deoxycholate and DNase. Similarly, Fernandez et al. (2016) reported a ∼92% reduction in dsDNA content and ∼30% retained sGAG content with their most effective detergent-based protocol for bovine NP, involving treatment with Triton-X100, DNase, and RNase. Hensley et al. (2018) applied a combination of detergents, ultrasonication, freeze-thaw cycles, and digestion with DNase and RNase to decellularize intact bovine IVDs, showing an ∼84% decrease in DNA content coupled with ∼55% retention of sGAGs within the NP region. The enhanced preservation of GAGs may be related to their strategy of processing of the tissues with the annulus fibrosus intact, as it may have functioned as a barrier to reduce GAG loss. Further extending the whole tissue approach, Norbertczak et al. (2020) subjected bovine IVDs that included intact cartilaginous endplates to a decellularization protocol involving SDS treatment, freeze-thaw cycles, sonication, and DNase/RNase digestion. There was no significant reduction in DNA content in the decellularized NP compared to the native NP controls, indicating that NP decellularization was impeded by presence of the surrounding tissues. Safranin O staining showed a marked reduction in staining intensity in the NP following decellularization, while DMMB assay results showed greater sGAG loss in the outer regions of the IVD that were more effectively decellularized.

Importantly, our immunohistochemical characterization studies confirmed that the DNP generated with our protocol also contained multiple collagen types as expected based on the native tissue source (Roughley, 2004), with intense staining for collagen type II, which is the most abundant collagen type within the healthy NP [∼15%–20% of the NP ECM dry weight (Hwang et al., 2014)]. Moreover, the cell-adhesive glycoproteins laminin and fibronectin were retained, which may be favorable for mediating cell adhesion, viability, and other downstream responses (Francisco et al., 2013; Hwang et al., 2014). Given the well characterized structural heterogeneity of the native NP in terms of ECM fragmentation (Craddock et al., 2018), future studies will focus on assessing the integrity of the ECM within the DNP in the context of ECM integrity and biological activity.

Following the establishment of the decellularization protocol, our next objective was to apply our expertise in ECM-derived bioscaffold fabrication to integrate the DNP into customized platforms tuned for 2-D and 3-D cell culture. As a starting point, we adapted methods that we previously established using adipose-derived ECM to generate novel DNP coatings (Shridhar et al., 2020). ECM-derived coatings from a variety of tissues have previously been shown to guide cell attachment, proliferation, and lineage-specific differentiation (DeQuach et al., 2010; Rothrauff, 2017; Shridhar et al., 2020). In contrast to the common strategy of digesting the ECM with the proteolytic enzyme pepsin, our approach involves digestion with α-amylase and homogenization in acetic acid to create an ECM suspension with preserved collagen fibrils. Digestion with α-amylase cleaves carbohydrate groups in the collagen telopeptide regions and can destabilize collagen-glycoprotein complexes, enhancing dispersion of the collagen fibrils within the acetic acid and allowing for the creation of more homogeneous coatings (Shridhar et al., 2020). Our previous study comparing coatings generated with α-amylase-digested DAT versus pepsin-digested DAT showed that α-amylase digestion generated coatings that were softer, thicker and more stable, and that retained a fibrous tissue-like ultrastructure that was completely lost following pepsin digestion (Shridhar et al., 2020). A similar multi-layer fibrous ultrastructure was observed in the current study in both the DNP and collagen control coatings.

Both the DNP and COL coatings were shown to support cell attachment and growth, with serial passaging enabled through collagenase digestion. However, neither coating type was able to maintain the NP phenotype following serial passages based on gene expression analysis. More specifically, consistent with previous findings with primary NP cells cultured on TCP (Cui et al., 2012; Rosenzweig et al., 2017; Ashraf et al., 2020), we observed a reduction in the mRNA expression levels of the NP-associated markers and type II collagen with serial passaging on TCP and COL coatings. The increased expression of *AGG* we detected in P0 NP cells cultured on COL coatings compared to TCP is consistent with previous reports of increased proteoglycan synthesis in rabbit NP cells cultured on collagen coatings (Inoue et al., 2005). Of note, the expression of the NP-associated markers *KRT8*, *KRT18*, and *SOX9* were reduced in P0 NP cells on DNP coatings compared to cells cultured on TCP and COL, demonstrating that the composition of the 2-D ECM coatings impacted cell phenotype. Additionally, the gene expression results indicated that the DNP coatings were unable to rescue the NP phenotype of de-differentiated P3 cells.

One possible limitation was that the further processing steps required for coating fabrication resulted in a significant reduction in GAG content relative to the DNP, with only ∼10% retention compared to the native NP tissues, which may have impacted the bioactivity of the coatings. In addition to the direct effects associated with loss of ECM proteoglycans, the loss of GAG content would indirectly impact additional factors, including the content of matricellular proteins and growth factors that serve as important biological regulators of the NP microenvironment (Bedore et al., 2014; Guerrero et al., 2021a). Moreover, we expect that the observed changes in ECM composition of the DNP within 2-D coatings would be associated with changes in both topography and stiffness of the coatings, which would alter the cellular response. In addition, we postulated that the 2-D nature of this platform may not have been favorable for maintaining the differentiated state, based on previous reports that culture of NP cells in 3-D is more effective at retaining the NP phenotype (Yuan et al., 2011; Guerrero et al., 2021b). One postulated factor that may be influencing this response is that NP cells can retain their rounded morphology when cultured in 3-D, while 2-D culture results in a spindle-shaped morphology (Yuan et al., 2011), as was observed in the current study.

As a next-step, we decided to investigate the response of the primary bovine NP cells encapsulated within 3-D MCS hydrogels incorporating DNP particles, which had significantly higher sGAG content as compared to the DNP coatings and were expected to promote a rounded NP cell shape. We selected MCS as it mimics the native proteoglycans of the NP, and have previously shown that high viability was maintained over 14 days in culture when human ASCs were encapsulated within the hydrogels, both with and without the incorporation of DAT or decellularized trabecular bone (DTB) particles (Shridhar et al., 2019). The addition of the DNP or COL particles at 5% w/v did not alter the crosslinking efficiency or bulk compressive properties of the hydrogels, supporting the application of the platforms specifically for exploring the effects of the ECM particles on the cellular response.

All three MCS hydrogel systems supported the viability of the encapsulated bovine NP cells. The composite MCS hydrogels incorporating the DNP or COL particles qualitatively contained a higher density of viable cells compared to the MCS only hydrogels following extended culture, consistent with our previous work that showed that the incorporation of DAT particles enhanced the retention of viable human ASCs encapsulated and cultured within the MCS hydrogels (Cheung et al., 2014). Cell clustering was observed at 7 and 14 days within the MCS + DNP and MCS + COL hydrogels, which we previously observed with the ASCs in the MCS + DAT composites (Cheung et al., 2014; Brown et al., 2015), suggesting that the ECM particles may have supported cell attachment. However, culture in the DNP- or COL-containing 3-D hydrogels did not prevent the loss of NP-associated gene expression in primary P0 cells observed over time in culture, nor restore the NP phenotype in de-differentiated P3 bovine NP cells.

One potential influencing factor is that the hydrogels were much stiffer than values reported for the native human NP of ∼5–25 kPa (Cloyd et al., 2007; Borrelli and Buckley 2020) and bovine NP of ∼6–19 kPa (Recuerda et al., 2011; Grenier et al., 2014). Notably, Gilchrist et al. (2011) demonstrated the importance of both ECM ligands and substrate stiffness on NP phenotype, with a more native NP morphology, enhanced cell-cell interactions, and augmented proteoglycan synthesis when porcine NP cells were cultured on soft (<720 Pa) ECM substrates that contained laminin. Further emphasizing the importance of mechanical properties, Xu et al. demonstrated that encapsulating rat NP cells within gelatin hydrogels of increasing stiffness resulted in increased cell de-differentiation (Xu et al., 2021). As such, the higher stiffness of the MCS hydrogels may have counteracted any beneficial effects of the DNP. In the future, it would be interesting to explore the cellular response within hydrogels that more closely mimic the native mechanical properties of the NP. The molecular weight of the chondroitin sulphate may have been another factor that influenced the cellular response based on the work of Jeong et al. (2014), whose biochemical assay results suggested that porcine NP cells cultured on the surface of hyaluronic acid (HA)-polyethylene glycol composite hydrogels synthesized with 27 kDa HA were undergoing de-differentiation, while those cultured on hydrogels synthesized with 59 kDa HA accumulated significantly more sGAG, although the mechanisms of these effects were unclear.

Strategies to enhance cell clustering within the hydrogels may be favorable for maintaining the NP phenotype within the hydrogels, mimicking the observed benefits of micromass cultures (Séguin et al., 2004; Gawri et al., 2017; Kushioka et al., 2019). Supporting this approach, Hwang et al. (2016) demonstrated that the formation of N-cadherin positive cell clusters was critical for maintaining the morphology, phenotype, and biosynthetic activity of human and porcine NP cells. Similarly, Spillekorn et al. (2014) showed that culturing canine notochord cells in native cell clusters within alginate hydrogels was favorable for retaining their phenotype in long-term culture.

While our culture models strive to mimic the native ECM composition, it is important to recognize that there are other factors within the cellular microenvironment that are key regulators of NP phenotype and function. While the ECM alone may be insufficient to maintain the differentiated phenotype of the bovine NP cells, it is possible that it could function synergistically with these other factors to have a beneficial effect. For example, while we previously observed only low levels of expression of adipogenic genes in the human ASCs cultured on the DAT coatings in proliferation medium, adipogenic gene and protein expression, as well as intracellular lipid accumulation, were significantly enhanced when the ASCs were cultured on the α-amylase-digested DAT coatings in adipogenic differentiation medium as compared to when the cells were cultured on α-amylase-digested COL coatings, pepsin-digested DAT or COL coatings, or TCP (Shridhar et al., 2020). Similarly, while there were no observed osteoinductive effects when the DTB particles were incorporated within the MCS hydrogels on encapsulated human ASCs, our previous findings support that the DTB had bioactive effects in modulating osteogenesis when combined with soluble factors within osteogenic differentiation medium (Shridhar et al., 2019). As such, supplementation of the medium with growth factors such as BMP-2 (Gilbertson et al., 2008), BMP-7 (Kawakami et al., 2005; Masuda et al., 2006; Wei et al., 2008), FGF-2 (Tsai et al., 2007), GDF-5, TGF-β1 (Henry et al., 2017) or TGF-β3, which have been shown to enhance NP cell survival and ECM synthesis (Ashraf et al., 2020), bioactive proteins such as CCN2 which has been shown to promote ECM synthesis in NP cells (Erwin et al., 2006), or SLRPs (e.g., biglycan or decorin) associated with enhanced survival and matrix synthesis in IVD progenitor cells (Chen et al., 2017), may help to maintain the NP phenotype in combination with the DNP and as such could be the focus of subsequent studies. In addition, it could be interesting to explore the effects of hypoxia, building from previous work that has shown that porcine NP cells better maintain their differentiated phenotype when cultured under 1% O_2_ as compared to atmospheric conditions (∼21% O_2_) (Jaworski et al., 2019). Finally, it would be interesting to explore the effects of mechanical stimulation on the phenotype of the NP cells encapsulated within our 3-D MCS hydrogels, which was also suggested by Priyadarshani et al. (2016), who similarly observed decreased expression levels of the NP-associated genes *SOX9*, *ACAN*, and *COL2A1*, coupled with increased expression of *COL1A1*, following long-term culture of human NP cells within type II collagen-HA composite hydrogels.

## 5 Conclusion

In the current study we successfully established a reproducible detergent-free decellularization protocol for bovine nucleus pulposus, and successfully integrated the DNP within novel 2-D and 3-D culture platforms. While the ECM alone was insufficient for maintaining the mature bovine NP phenotype based on analysis of gene expression, both platforms were shown to support cell viability in culture and further investigation of their utility is warranted to assess their potential as tissue-specific cell culture platforms for NP regenerative applications. Building from this work, it would be interesting to assess the phenotypic response at the protein level using techniques such as western blotting, ELISA, or immunohistochemistry, to see if there may be differences in matrix synthesis or secretion that were not detected at the gene expression level. Furthermore, it would be worthwhile to investigate alternative cell sources such as bone marrow-derived mesenchymal stromal cells (MSCs), adipose-derived stromal cells, or induced pluripotent stem cells (iPSCs), to see if the ECM could be harnessed within strategies to drive their lineage-specific differentiation towards an NP phenotype.

## Data Availability

The original contributions presented in the study are included in the article/Supplementary Materials, further inquiries can be directed to the corresponding authors.
