# Supplementary material for: Development of 2-D and 3-D culture platforms derived from decellularized nucleus pulposus
Source: Front Bioeng Biotechnol. 2022 Sep 27;10:937239. doi: 10.3389/fbioe.2022.937239 (PMC9551564; doi:10.3389/fbioe.2022.937239)
Supplement: Supplementary file 1 [file DataSheet1.docx]

Supplementary Material


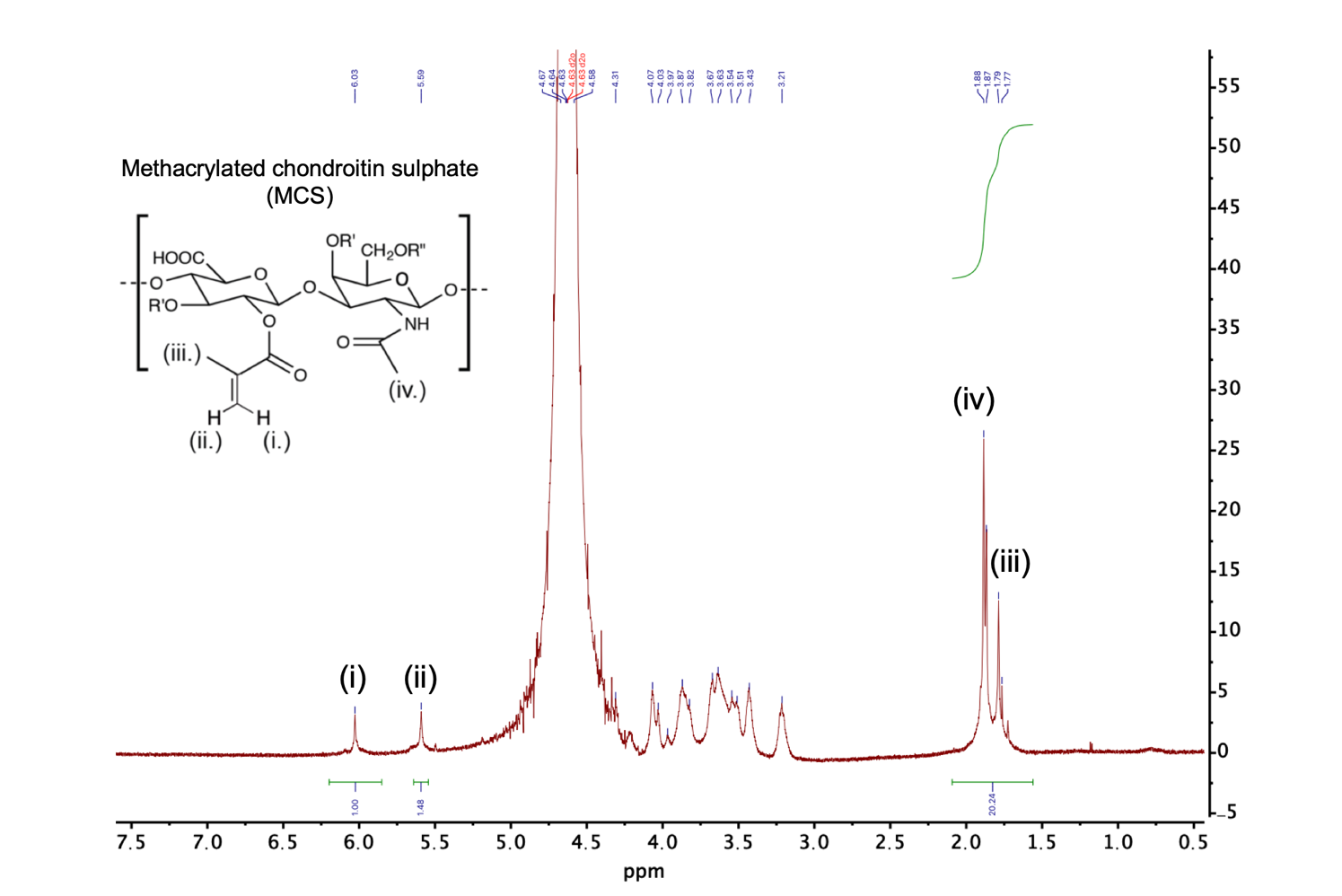


**Supplementary Figure 1*.* Representative 1H-NMR spectra of methacrylated chondroitin sulphate (MCS)*.*** Key peaks were identified and correlated to the chemical structure of the MCS polymer, comprised of repeating units of D-glucuronic acid and N-acetyl galactosamine. Peaks at ~6.1 ppm (i) and ~5.65 ppm (ii) correspond to the vinyl groups on the grafted methacrylate functional group. The peak at ~1.85 ppm (iv) corresponds to the methyl group protons on the methacrylate that was functionalized to the chondroitin sulphate. The peak at ~1.95 ppm (iii) corresponds to the proton on the methyl group of the native N-acetyl residues on the chondroitin sulphate. Integration of the protons confirmed a target degree of methacrylation of 17%. The absence of non-specific peaks in the spectra confirmed the absence of chemical contaminants in the purified MCS polymer. Spectra is representative of N=8 polymer batches.


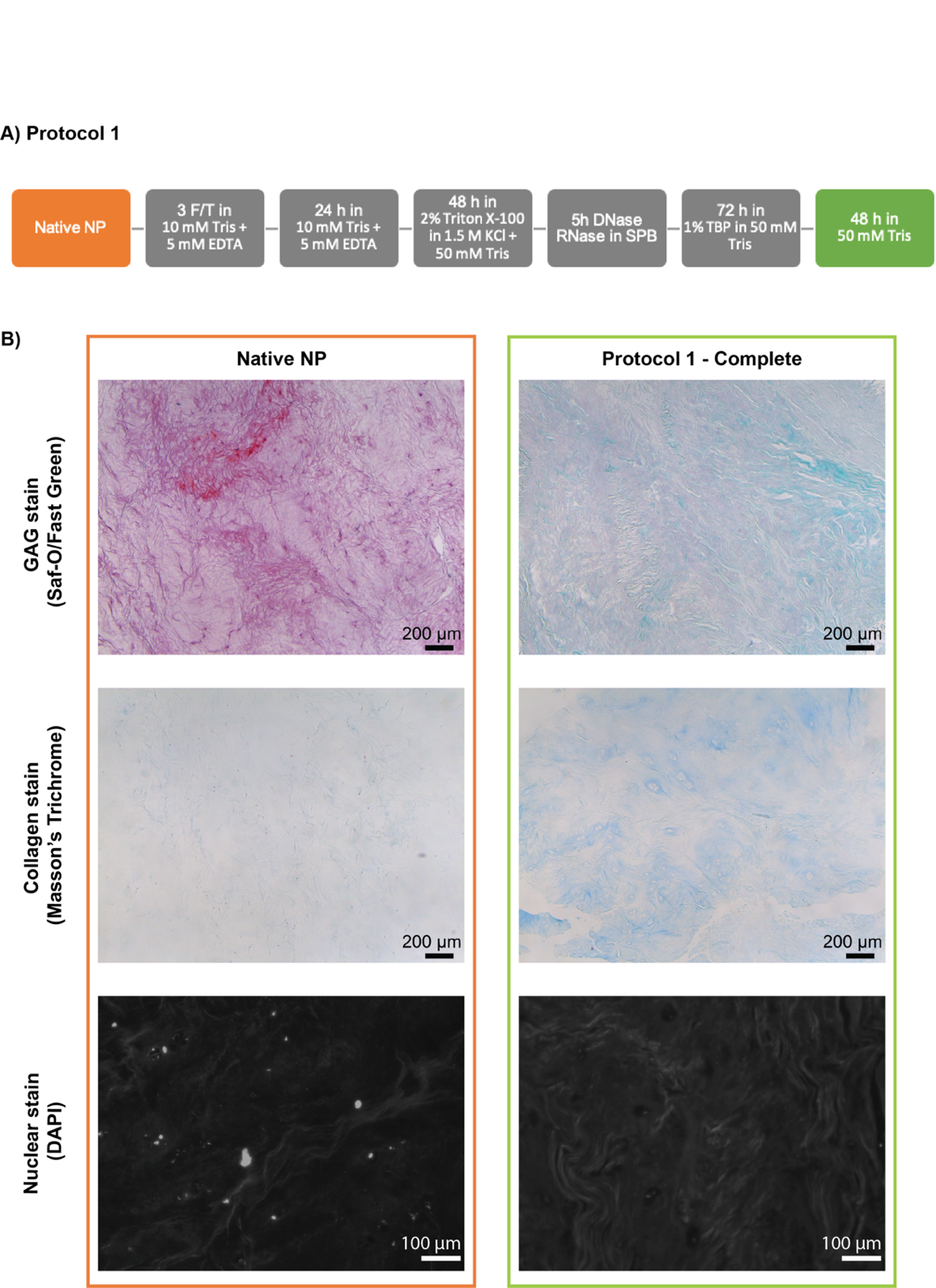


Supplementary Figure 2. Histological analyses of bovine NP tissue processed with the initial decellularization protocol. (A) Protocol overview, with sampling points shown in colour. Acronyms: F/T=freeze-thaw cycles, Tris=tris (hydroxymethyl)aminomethane, EDTA=ethylenediaminetetraacetic acid, SPB=Sorensen’s phosphate buffer, TBP=tributyl phosphate. (B) Representative safranin-O/fast green staining of GAG (red) and collagen (blue-green), showing minimal GAG retention in the DNP. Masson’s trichrome showing more intense staining of collagen in the DNP samples (blue). DAPI nuclear staining showing no detectable nuclei at the end of processing (white) (n=3, serial sections throughout the tissue sample). Scale bars for safranin-O/fast green and Masson’s trichrome = 200 µm. Scale bars for DAPI staining = 100 µm.

**
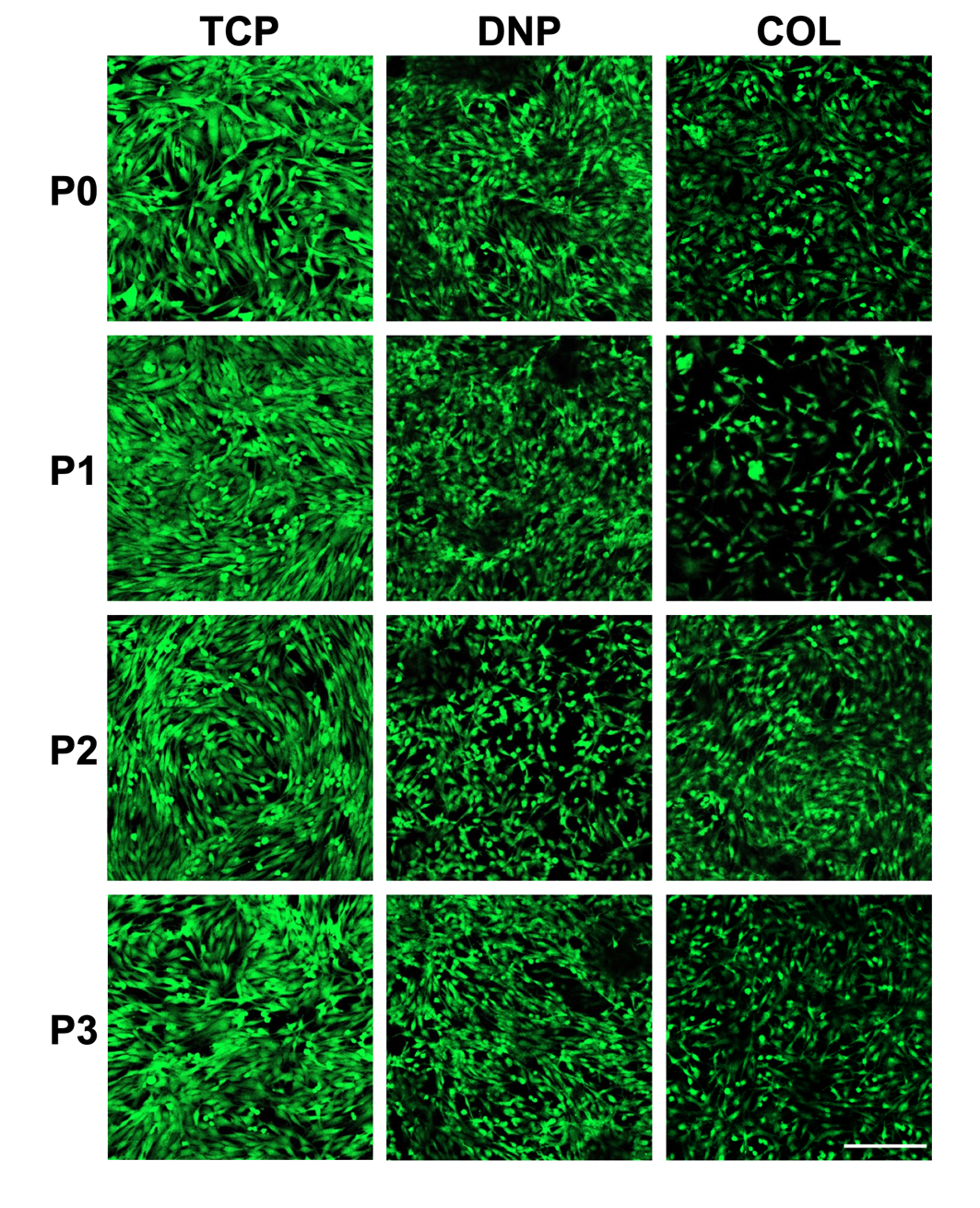
**

**Supplementary Figure 3. ECM coatings provide a supportive platform for primary bovine NP cell expansion *in vitro*.** Representative confocal microscopy images showing calcein-AM^+^ live primary bovine NP cells (green) following monolayer culture (P0) and serial passaging (P1-P3) on uncoated tissue culture plastic (TCP), decellularized NP (DNP) coatings, or type I collagen (COL) coatings. Images are representative of N=3 individual cell preparations. Scale bar = 200 μm.

**
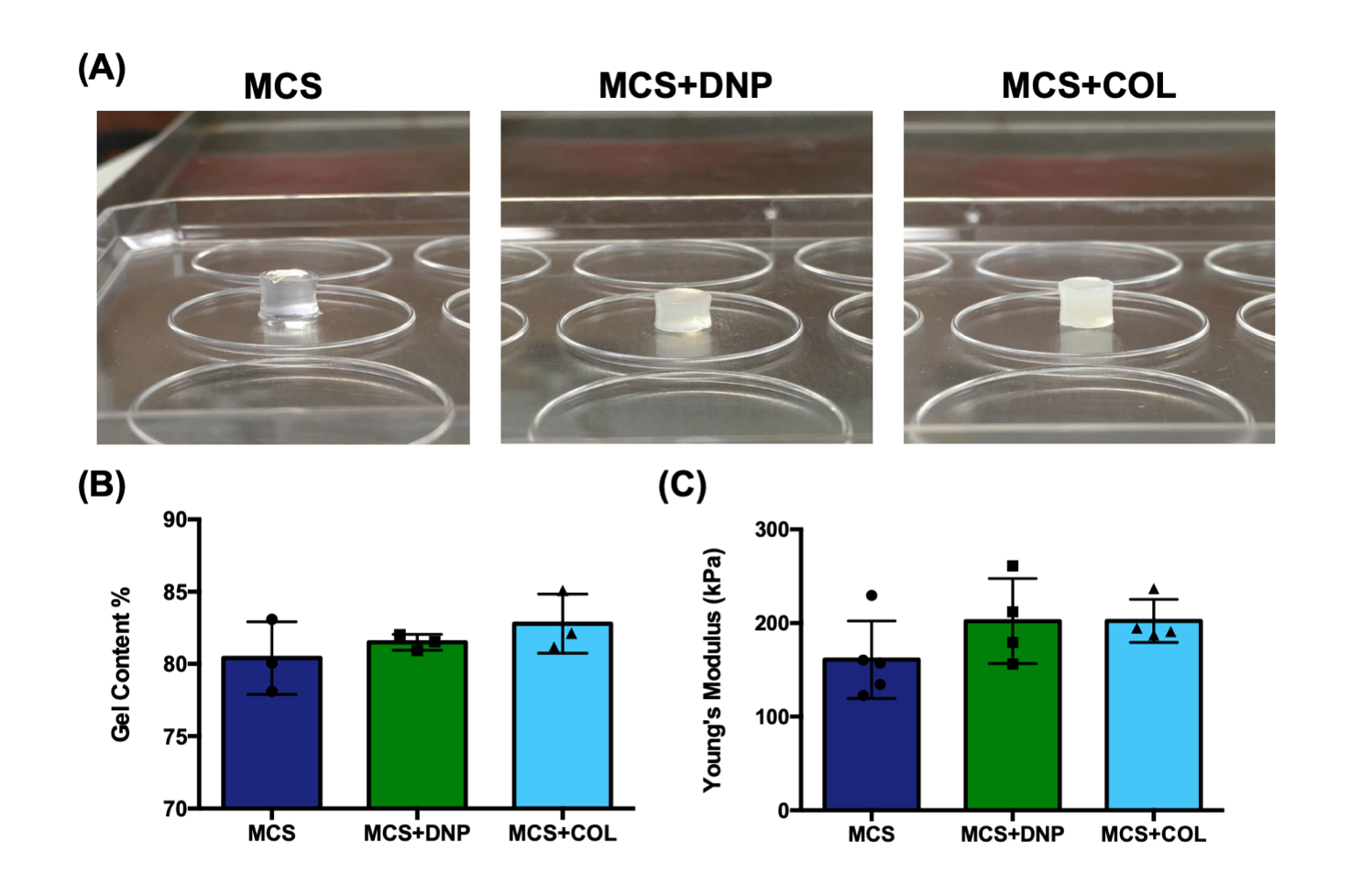
**

**Supplementary Figure 4. Characterization of MCS ± DNP/COL hydrogels. (A)** Macroscopic images of MCS ± DNP/COL hydrogels following UV-crosslinking and overnight equilibration in PBS at 37 °C. MCS hydrogels appeared transparent, while the MCS + DNP and MCS + COL hydrogel composites were opaque with an off-white colour. No significant differences were observed between the hydrogel groups in terms of **(B)** gel content analysis (n=5 individual hydrogels/trial, N=3 independent trials) or **(C)** Young’s moduli as determined through unconfined bulk compression testing (n=2-3 individual hydrogels/trial, N=4-5 independent trials). These data confirm that incorporation of the ECM particles did not interfere with crosslinking (p<0.05).

**
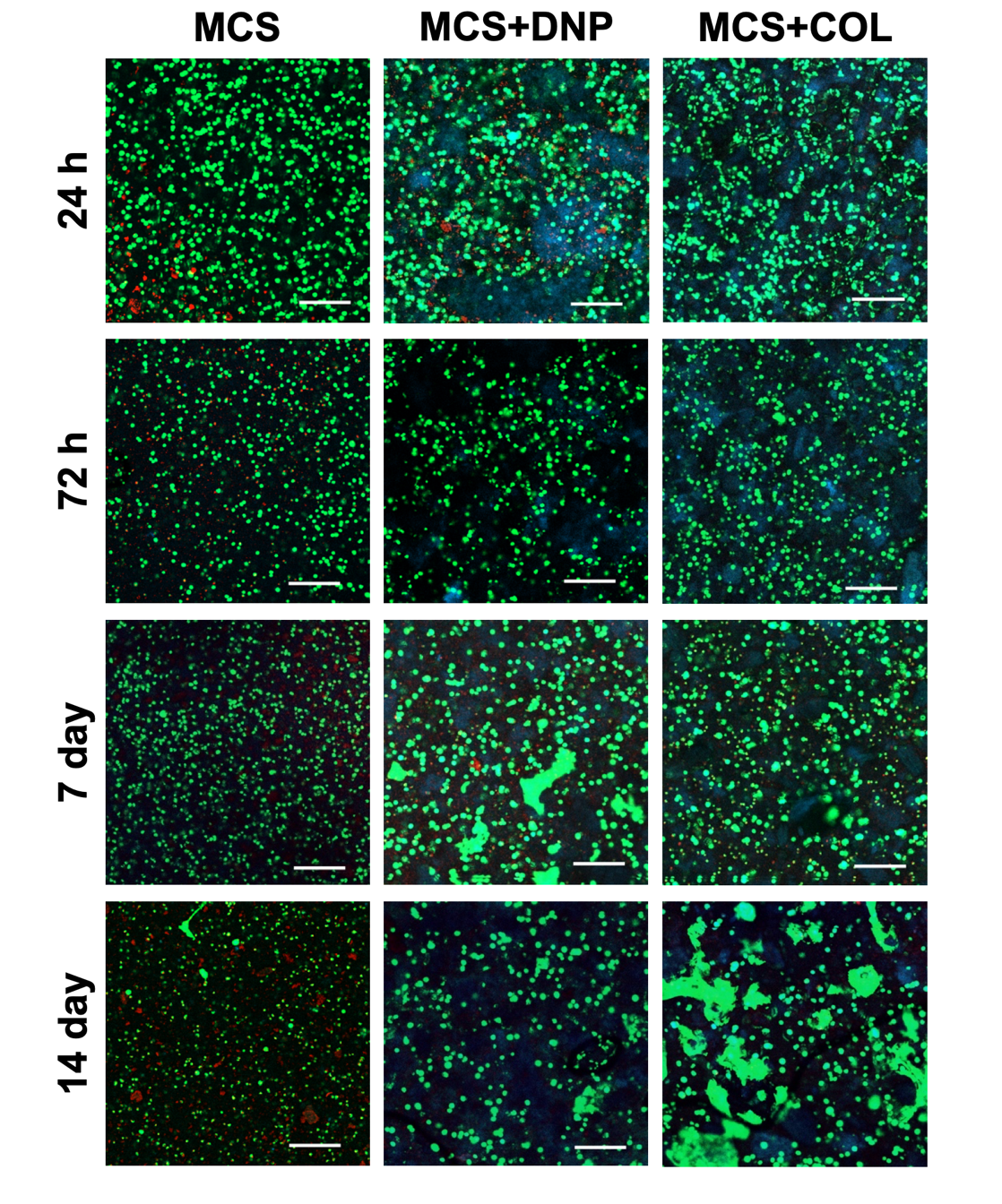
**

**Supplementary Figure 5. Primary passaged bovine NP cells remained viable following encapsulation and *in vitro* culture within MCS** ± **DNP/COL up to 14 days.** Late passage P3 primary bovine NP cells were encapsulated within MCS, MCS+DNP or MCS+COL hydrogels, and cultured *in vitro* for 24 h, 72 h, 7 days, or 14 days. Representative confocal microscopy images were captured at each time point showing live cells stained with calcein-AM (green), dead cells with ethidium homodimer-1 (red), and auto-fluorescence of DNP and COL particles (blue). Images were captured at depths between 75-150 μm from the surface of each hydrogel construct. Scale bars = 200 μm. Images are representative of N=3 individual cell preparations.
